# Therapeutic Effects and Mechanisms of the Inhaled Traditional Chinese Medicine Compound ZHW on Allergic Rhinitis

**DOI:** 10.3390/ph18071059

**Published:** 2025-07-18

**Authors:** Yujin Shen, Xi Ma, Zhenzhen Du, Yang Li, Zhinan Mei, Ling Zhao

**Affiliations:** School of Biology and Pharmaceutical Engineering, Wuhan Polytechnic University, Wuhan 430023, China; shenyujin04282022@126.com (Y.S.); maxiwhpu@126.com (X.M.); 18993988787@163.com (Z.D.); yangli@whpu.edu.cn (Y.L.); meizhinan@163.com (Z.M.)

**Keywords:** allergic rhinitis, ZHW, inhalation administration, proteomics, PI3K-Akt, FcεRI

## Abstract

**Background:** Allergic rhinitis (AR) is a prevalent allergic disorder characterized by a complex pathogenesis. Drawing on traditional Chinese medicine theory and contemporary pharmacological principles, this study developed an inhalation-based herbal formulation, ZHW, to explore a novel non-invasive therapeutic approach. **Objective:** To investigate the therapeutic effects of ZHW on AR and elucidate its underlying mechanisms and potential targets through an integrated analysis of network pharmacology and proteomics. **Materials and Methods:** The volatile components of ZHW were analyzed by gas chromatography–mass spectrometry (GC-MS). The mouse model of AR was induced by OVA sensitization. The therapeutic efficacy of ZHW was assessed based on nasal symptom scores, histopathological examination, and inflammatory cytokine levels. Furthermore, the underlying mechanisms and potential targets of ZHW were investigated through integrated network pharmacology and proteomics analyses. **Results:** GC-MS analysis identified 39 bioactive compounds in ZHW. Inhalation treatment with ZHW demonstrated significant anti-allergic effects in OVA-sensitized mice, as evidenced by (1) reduced sneezing frequency and nasal rubbing behaviors; (2) decreased serum levels of IL-4, histamine, and OVA-specific IgE; (3) attenuated IL-4 concentrations in both nasal lavage fluid and lung tissue; (4) diminished nasal mucosal thickening; and (5) suppression of inflammatory cell infiltration. Integrated network pharmacology and proteomics analyses indicated that ZHW’s therapeutic effects were mediated through the modulation of multiple pathways, including the PI3K-Akt signaling pathway, the B cell receptor signaling pathway, oxidative phosphorylation, and the FcεRI signaling pathway. Key molecular targets involved Rac1, MAPK1, and SYK. Molecular docking simulations revealed strong binding affinities between ZHW’s primary bioactive constituents (linalool, levomenthol, linoleic acid, Linoelaidic acid, and n-Valeric acid cis-3-hexenyl ester) and these target proteins. **Conclusions:** The herbal formulation ZHW demonstrates significant efficacy in alleviating allergic rhinitis symptoms through multi-target modulation of key signaling pathways, including PI3K-Akt- and FcεRI-mediated inflammatory responses. These findings substantiate ZHW’s therapeutic potential as a novel, non-invasive treatment for AR and provide a strong basis for the development of new AR therapies. Future clinical development will require systematic safety evaluation to ensure optimal therapeutic outcomes.

## 1. Introduction

As a type I hypersensitivity reaction, allergic rhinitis (AR) manifests through nasal inflammation, causing characteristic symptoms: congestion, rhinorrhea, pruritus, and repetitive sneezing [1]. Recent epidemiological studies have demonstrated a significant increase in the prevalence of AR globally. In China, approximately 190 million adults and 220 million children suffer from AR, accounting for 13.26% and 15.79% of the total population, respectively, while the global prevalence is as high as 10–40% [2].

The pathogenesis of AR is complex, primarily involving IgE-mediated type I hypersensitivity reactions and a Th1/Th2 cell imbalance [3]. Upon initial allergen exposure, T cell activation occurs, which in turn stimulates B lymphocytes to generate allergen-specific IgE antibodies. These immunoglobulins subsequently bind to the surface receptors of mast cells (MCs) and eosinophils (EOSs), completing sensitization. When the allergens reappear, they cross-link with IgE antibodies, causing degranulation and the release of inflammatory mediators such as histamine, thereby inducing acute allergic symptoms [3,4].

The pathogenesis of allergic rhinitis involves a characteristic Th1/Th2 immune imbalance. While Th1-derived IFN-γ maintains cellular immune responses, Th2 cytokines (such as IL-4, IL-5, and IL-13) drive the allergic cascade through IgE class-switching and eosinophil recruitment/activation [5]. In AR patients, Th2 cell overactivation elevates IgE levels, exacerbating allergic responses. Additionally, Th2 cytokines facilitate eosinophil and mast cell infiltration in the nasal mucosa, further amplifying inflammation. Therefore, restoring Th1/Th2 balance—particularly by suppressing excessive Th2 activation—has become a key therapeutic strategy for AR [6].

Currently, the treatment options for AR include patient education, avoidance of irritants, pharmacotherapy, allergen immunotherapy, nasal irrigation, acupuncture, and surgery [7]. Compared to Western medications that often cause undesirable side effects during prolonged treatment, traditional Chinese medicine (TCM) has demonstrated distinct advantages in AR management through its long-standing clinical experience and holistic approach. Classical prescriptions such as Cang-Er-Zi-San [8], Xiao-Qing-Long-Tang [9], and Yu-Ping-Feng-San [10] can effectively improve AR symptoms with high safety and fewer adverse reactions. Some TCM formulations have also been proven to alleviate inflammatory responses by inhibiting Th2 cell activation. For example, Liu et al. [8]. demonstrated that Cang-Er-Zi-San effectively relieves allergic symptoms in AR patients with adenoid hypertrophy; Liu et al. [9] confirmed the stability and safety of active components in Xiao-Qing-Long-Tang and showed significant downregulation of Th2 inflammatory cytokines in AR mice; Tong et al. [10] further proved that Yu-Ping-Feng-San can ameliorate pathological changes in the nasal mucosa of OVA-induced AR mice and reduce the production of inflammatory and immune factors.

With the deepening of clinical practice, modifications based on classical prescriptions could enhance therapeutic efficacy and optimize drug delivery methods. Inhalation therapy offers significant advantages in the treatment of rhinitis, as its volatile active components can directly act on the nasal mucosa, ensuring the rapid onset of action while bypassing hepatic first-pass metabolism, making it particularly suitable for pediatric and elderly patients. Cang-Er-Z-San, a classical TCM formula composed of *Xanthium sibiricum* Patr., *Magnolia denudata* Desr., *Angelica dahurica*, and *Mentha haplocalyx* Briq. [8] is widely used for nasal disorders with a clinical efficacy rate as high as 95.74% [11]. Building upon this foundation, our study developed a novel ZHW compound herbal granule for heated inhalation therapy by incorporating *Houttuynia cordata* Thunb. and *Citrus reticulata* Blanco. The addition of *Houttuynia cordata* Thunb. enhances anti-inflammatory effects, while *Citrus reticulata* Blanco. contributes an aromatic profile to improve compliance. To elucidate the pharmacological basis of ZHW, we employed GC-MS to analyze its volatile components and utilized network pharmacology to predict potential interactions with AR-related targets. Furthermore, we established an OVA-sensitized Kunming mouse model of AR and applied proteomics to evaluate the therapeutic effects and underlying mechanisms of ZHW. This study provides a scientific foundation for novel AR treatments and advances the modern application of traditional Chinese medicine.

## 2. Results

### 2.1. ZHW Compounds

In the volatile oil of ZHW, a total of 257 compounds were detected. However, some of these compounds were present in relatively low amounts. In this study, only compounds with a relative content of more than 0.2% and a matching degree greater than 80% were selected. Ultimately, 39 compounds were identified, which accounted for approximately 82.84% of the total components (Table 1).

### 2.2. Network Pharmacology Analysis of ZHW

#### 2.2.1. Database Retrieval and Target Prediction

**Figure 1:**
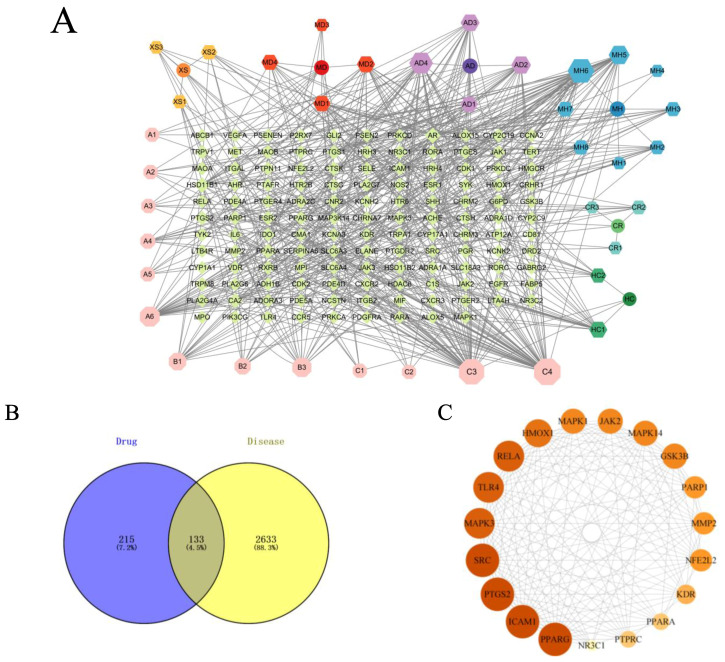

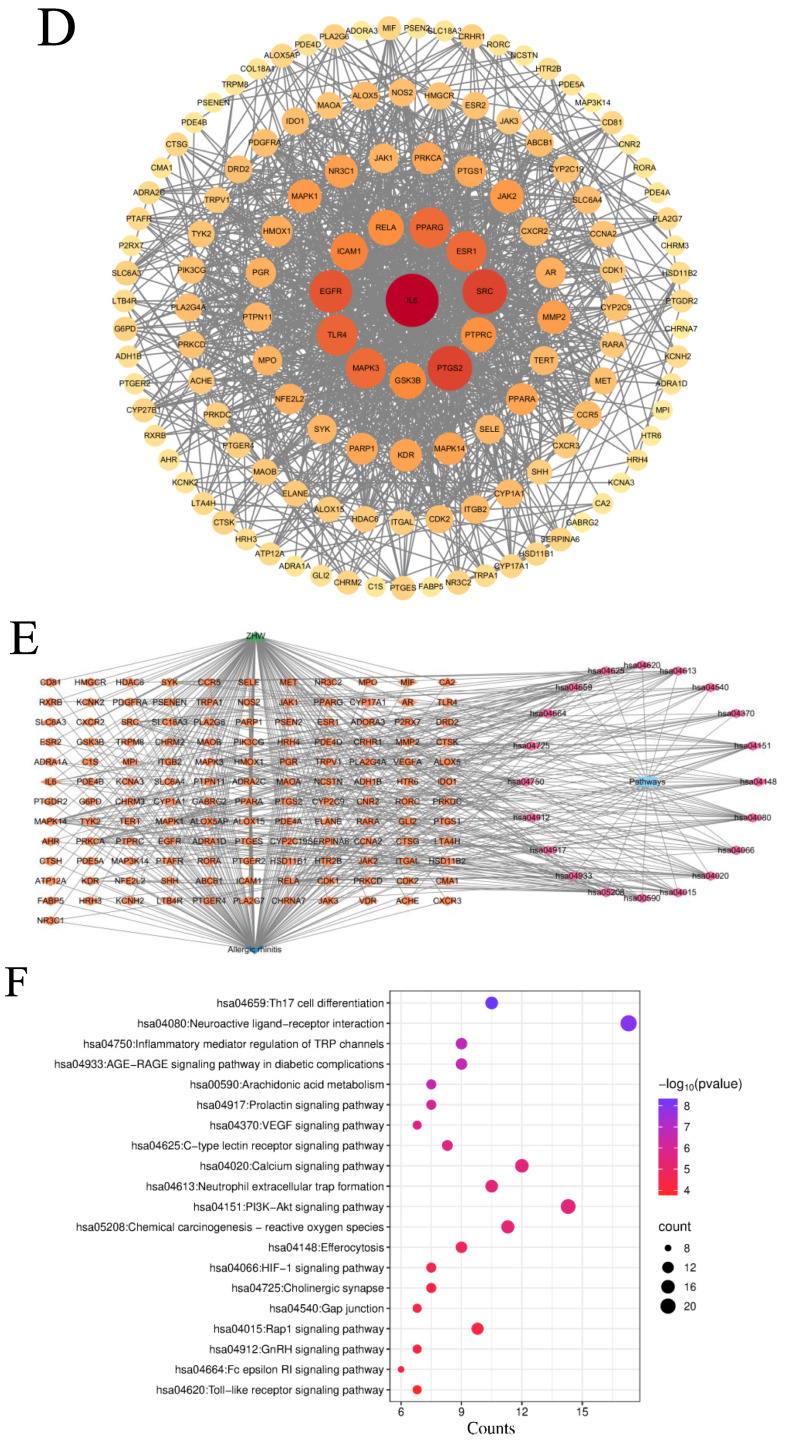

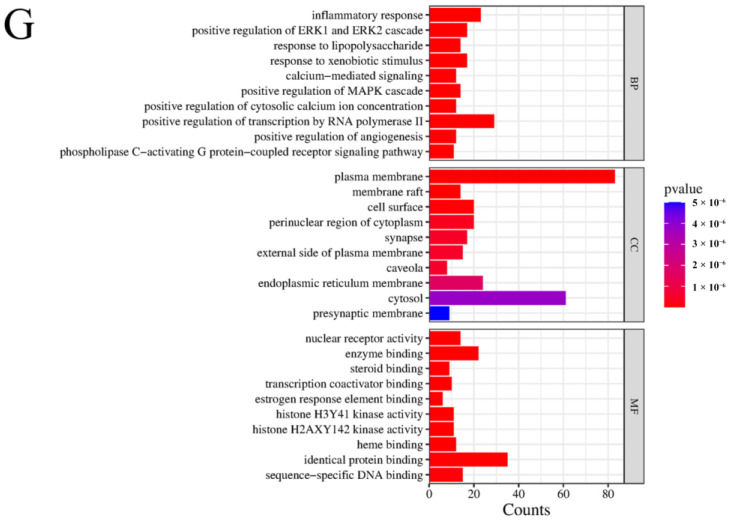
Results of network pharmacology analysis. (**A**) Signaling pathways and targets of ZHW related to the anti-allergic rhinitis effect. The circular nodes represent all herbal components of ZHW, each encircled by their respective bioactive compounds. Color-coded hexagonal nodes denote individual ZHW compounds, while light pink octagonal nodes indicate shared constituents across multiple herbs. Gene targets are displayed as green rhombus nodes arranged in a rectangular matrix, with target degree values visually encoded through node size variation. (**B**) Common targets between ZHW and anti-allergic rhinitis effect. (**C**) Interactions among core target proteins. (**D**) Correlation between common targets in the PPI network. (**E**) Target pathway network. (**F**) Results of the KEGG enrichment analyses. (**G**) Results of the GO enrichment analyses.

The 2766 disease targets obtained from the database were integrated with the 348 targets acquired from the 39 components, ultimately yielding 133 intersecting targets, as shown in Figure 1B.

#### 2.2.2. Construction of Component–Target Network

To visually demonstrate the interactions between ZHW compounds and their potential targets, we imported the compound–target data into Cytoscape 3.10.0 software (https://www.cytoscape.org, accessed on 20 November 2023.) to construct a network diagram of ZHW active compounds and their targets (Figure 1A). This network diagram illustrates that the active components of ZHW are associated with the anti-allergic rhinitis effect. Among them, the top three core components with the highest number of connections are C3, linoleic acid, C4, trans-linoleic acid, and MH6, pentyl pentanoate.

#### 2.2.3. Protein–Protein Interaction Network Analysis

The protein interaction network of the 133 potential targets of ZHW for the treatment of AR was analyzed using the STRING database (Figure 1D). The core network of the protein network was screened using the cytoHubba plugin of Cytoscape 3.10.0 software to obtain the top 19-protein core network. The top ten core targets include PPARG, ICAM1, PTGS2, SRC, MAPK3, TLR4, RELA, HMOX1, MAPK1, and JAK2 (Figure 1C).

#### 2.2.4. Pathway Enrichment Analysis

KEGG enrichment analysis (Figure 1F) was employed to predict the mechanisms underlying the improvement of allergic rhinitis mediated by ZHW, and an intersecting target pathway network was constructed (Figure 1E). The results showed that the top 20 KEGG-enriched signaling pathways included Th17 cell differentiation, regulation of inflammatory mediators by TRP channels, arachidonic acid metabolism, the PI3K-Akt signaling pathway, the FcεRI signaling pathway, and the Toll-like receptor signaling pathway, among others. Additionally, KEGG enrichment analysis revealed key signaling pathways associated with allergic rhinitis, such as the B cell receptor signaling pathway, IL-17 signaling pathway, MAPK signaling pathway, and NF-κB signaling pathway. These findings suggest that the potential mechanisms of ZHW as an anti-allergic rhinitis drug may be closely related to the PI3K-Akt signaling pathway and the FcεRI signaling pathway.

#### 2.2.5. Gene Ontology (GO) Function Enrichment Analysis

The GO enrichment analysis (Figure 1G) revealed that the biological processes (BPs) involved included the inflammatory response, lipopolysaccharide response, response to exogenous stimuli, and calcium-mediated signaling pathways. The cellular components (CCs) included the plasma membrane, membrane rafts, the cell surface, and the perinuclear region of the cytoplasm. The molecular functions (MFs) were primarily associated with nuclear receptor activity, enzyme binding, steroid binding, and homodimerization.

### 2.3. Effects of ZHW on Body Weight and Behavior in OVA-Induced AR Mice

**Figure 2:**
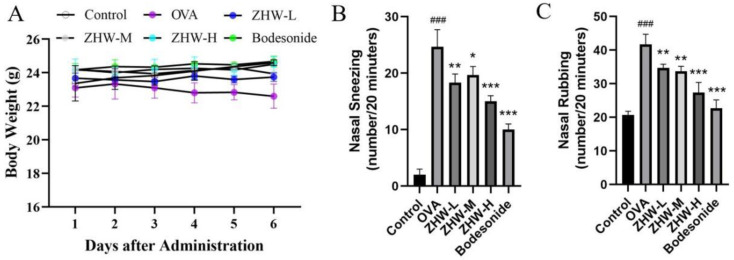
Effects of ZHW on the body weight and nasal symptoms of OVA-induced AR mice. (**A**) Mouse body weight. (**B**) Number of sneezes (within 20 min). (**C**) Number of nose scratches (within 20 min). The values shown in the figure are mean ± SD (n = 9). ANOVA with the Bonferroni post hoc test; compared with the control group, ### *p* < 0.001. Compared with the OVA group, * *p* < 0.05; ** *p* < 0.01; *** *p* < 0.001.

During the experimental period, the changes in body weight of the mice in each group were regularly monitored to evaluate the impact of ZHW on their growth and development. The results showed no significant differences in body weight among the control group, OVA group, ZHW-L group, ZHW-M group, and ZHW-H group (Figure 2A). This indicates that ZHW has no obvious toxic effects on body weight or food intake in mice. Compared with the control group, the number of sneezing (Figure 2B) and nose scratching (Figure 2C) episodes in the OVA-induced AR group was significantly increased (*p* < 0.001). All three ZHW treatment groups (low-dose group at 0.5 g/dose, medium-dose group at 1.0 g/dose, and high-dose group at 2.0 g/dose) showed significant reductions in sneezing and nose-scratching behaviors. These results suggest that inhalation of ZHW can effectively alleviate OVA-induced allergic rhinitis symptoms in vivo.

### 2.4. Effects of ZHW on Immune Function and Peripheral White Blood Cell Counts in OVA-Induced Mice

**Figure 3:**
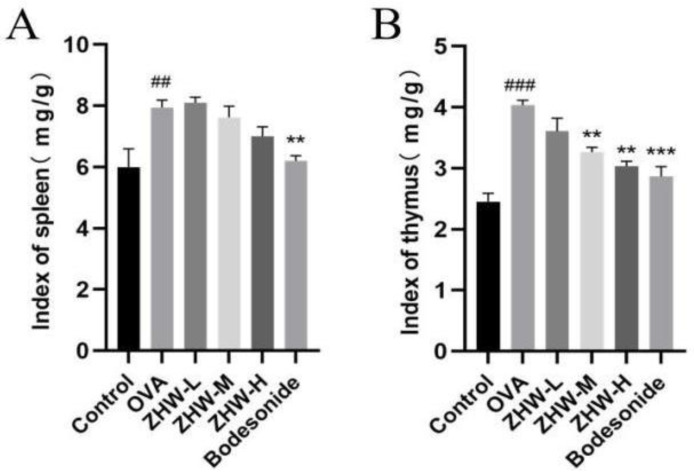
Effects of ZHW on the spleen and thymus indices of OVA-induced AR mice. (**A**) Spleen index (mg/g). (**B**) Thymus index (mg/g). The values shown in the figure are mean ± SD. (n = 9). ANOVA with Bonferroni post hoc test; compared with the control group, ## *p* < 0.01; ### *p* < 0.001. Compared with the OVA group, ** *p* < 0.01; *** *p* < 0.001.

The spleen and thymus indices of the mice in each group were measured. The spleen and thymus are important immune organs of the body, and changes in their weights can reflect the immune status of the body. The experimental results showed that, compared with the control group, the spleen and thymus indices of the OVA group were significantly increased (*p* < 0.01), indicating that the OVA-induced AR mice were in an immune-activated state (Figure 3A,B). Following ZHW intervention, both spleen and thymus indices showed varying degrees of reduction, with the medium- and high-dose ZHW groups exhibiting significant decreases in the thymus index (*p* < 0.01), suggesting that ZHW can regulate the weight of immune organs and exert a certain regulatory effect on the immune system.

**Figure 4:**
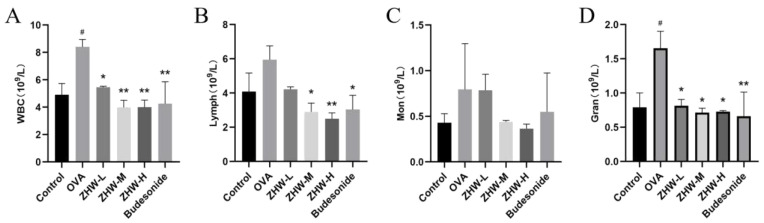
Effects of ZHW on peripheral blood leukocyte counts in OVA-induced AR mice. (**A**) WBC (total white blood cells). (**B**) Lymph (peripheral blood lymphocytes). (**C**) Mon (monocytes). (**D**) Gran (eosinophils). The values shown in the figure are mean ± SD (n = 9). ANOVA with Bonferroni post hoc test; compared with the control group, # *p* < 0.05. Compared with the OVA group, * *p* < 0.05; ** *p* < 0.01.

Additionally, as shown in Figure 4, compared with the control group, the OVA group had increased levels of WBC and Gran (*p* < 0.05). After treatment with heated ZHW inhalation, there was a significant reduction in WBCs, Lymph, and Gran, with these changes being dose-dependent. However, there were no significant differences in Mon among the groups (*p* > 0.05).

### 2.5. Effects of ZHW on Serum IFN-γ, IL-4, HIS, and OVA-sIgE Levels in OVA-Induced AR Mice

**Figure 5:**
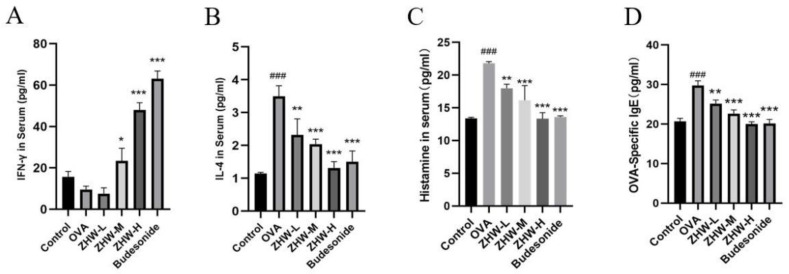
Effects of ZHW on serum IFN-γ, IL-4, HIS, and OVA-sIgE in OVA-induced AR mice. (**A**) IFN-γ. (**B**) IL-4. (**C**) HIS. (**D**) OVA-sIgE. The values shown in the figure are mean ± SD (n = 9). ANOVA with Bonferroni post hoc test; compared with the control group, ### *p* < 0.001. Compared with the OVA group, * *p* < 0.05; ** *p* < 0.01; *** *p* < 0.001.

To evaluate the therapeutic effects of ZHW on AR, we measured the levels of Th1- and Th2-related cytokines in NALF using ELISA. IFN-γ, a Th1-type cytokine, has antiviral, antitumor, and immune regulatory functions [12]. As illustrated in Figure 5A, following ZHW treatment, the serum levels of IFN-γ in the medium- and high-dose groups were significantly elevated. This suggests that ZHW is capable of effectively modulating the Th1-type immune response and bolstering the body’s capacity to combat infections.

IL-4, a Th2-type cytokine, promotes the proliferation and differentiation of B cells and the production of IgE [13]. The experimental results showed that the serum IL-4 levels in the OVA group were significantly higher than those in the control group (*p* < 0.001), indicating that the OVA-induced AR mice were in a state of overactivated Th2-type immune response (Figure 5B). After ZHW treatment, the serum IL-4 levels in mice of all dose groups were significantly reduced in a dose-dependent manner (*p* < 0.01), indicating that ZHW can effectively inhibit the Th2-type immune response and alleviate AR symptoms.

Histamine (HIS) is an important inflammatory mediator that can cause vasodilation, smooth muscle contraction, and increased mucus secretion, and is one of the main inducers of AR symptoms [14]. OVA-specific IgE (OVA-sIgE) is a key factor in the pathogenesis of AR [15], which can recognize and bind to allergens to trigger IgE-mediated allergic reactions. Compared with the control group, the levels of HIS (Figure 5C) and OVA-sIgE (Figure 5D) in the serum of the OVA-induced AR group were significantly increased. After ZHW treatment, the levels of HIS and OVA-sIgE in the serum of mice in all dose groups were significantly reduced in a dose-dependent manner. These data suggest that ZHW may improve OVA-induced AR symptoms by increasing the levels of IFN-γ and inhibiting the secretion of IL-4, thereby regulating the balance between Th1 and Th2, and suppressing the release of HIS and OVA-sIgE in the body.

### 2.6. Effects of ZHW on IFN-γ and IL-4 Levels in Lung Tissue and NALF of OVA-Induced AR Mice

**Figure 6:**
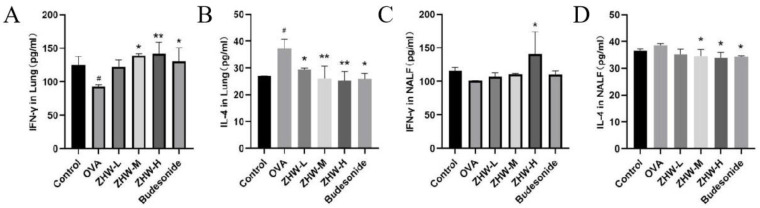
Effects of ZHW on IFN-γ and IL-4 in lung and nasal lavage fluid (NALF) of OVA-induced AR mice. (**A**) IFN-γ in lung. (**B**) IL-4 in lung. (**C**) IFN-γ in NALF. (**D**) IL-4 in NALF. The values shown in the figure are mean ± SD (n = 9). ANOVA with Bonferroni post hoc test; compared with the control group, # *p* < 0.05. Compared with the OVA group, * *p* < 0.05; ** *p* < 0.01.

To further investigate the local immune regulatory effects of ZHW on OVA-induced AR mice, the levels of IFN-γ and IL-4 in lung tissue and NALF were measured. These two cytokines play a key role in the pathogenesis of AR and can reflect the state of local immune responses [16]. As shown in Figure 6A, OVA induction significantly reduced the levels of IFN-γ in the lung tissue of AR mice, indicating suppression of the Th1-type immune response, while the levels of IL-4 were significantly increased, indicating overactivation of the Th2-type immune response (Figure 6B). After ZHW treatment, the levels of IFN-γ in lung tissue were significantly increased, and the levels of IL-4 were significantly decreased. Additionally, as shown in Figure 6C,D, there were no significant differences in the levels of IFN-γ and IL-4 in the NALF of OVA-induced AR mice compared with the control group. However, after treatment with a high dose of ZHW (ZHW-H), the levels of IFN-γ in NALF were significantly increased (*p* < 0.05), and the levels of IL-4 were significantly decreased (*p* < 0.05). These results suggest that ZHW-H may help regulate the balance of Th1 and Th2 cytokines locally in nasal tissues, contributing to the alleviation of AR symptoms.

### 2.7. Effects of ZHW on Nasal Mucosa and Lung Tissue Pathological Changes in Mice

**Figure 7:**
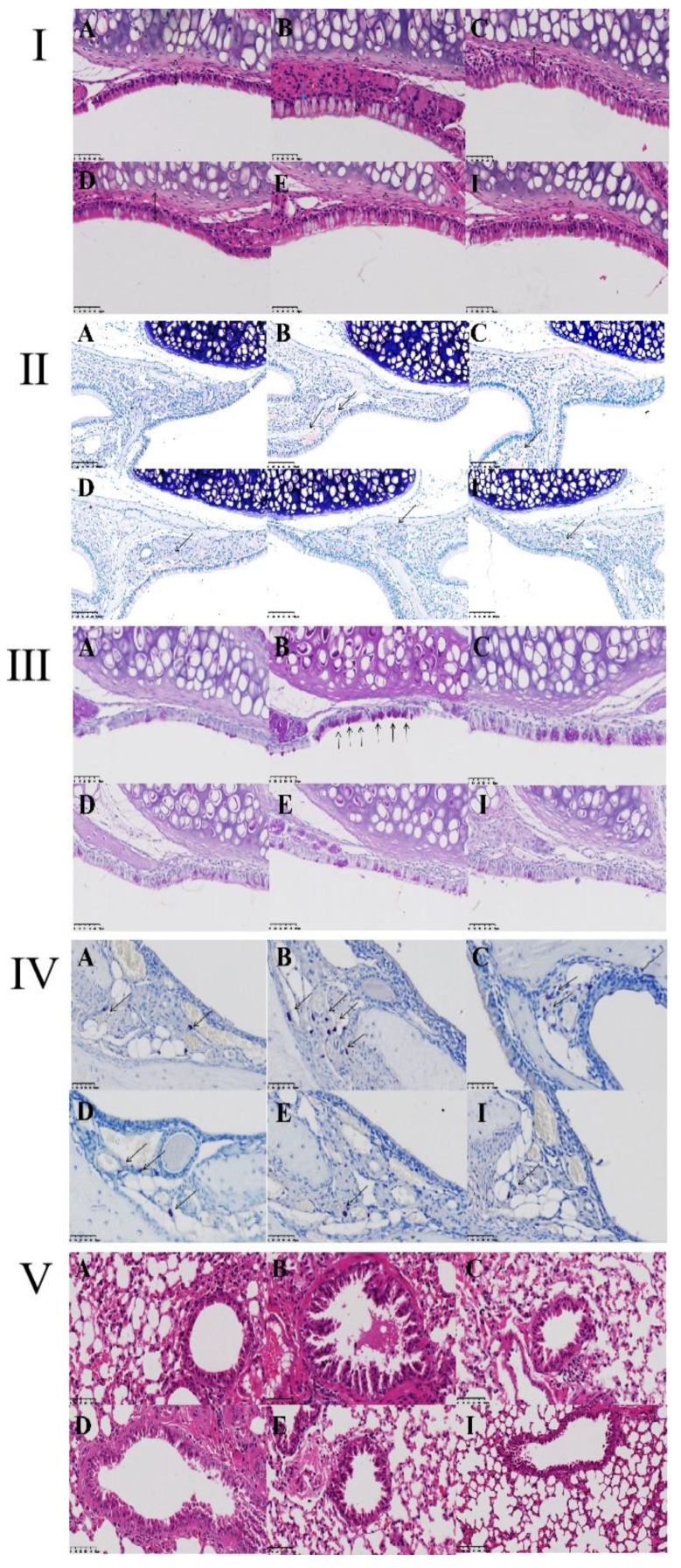

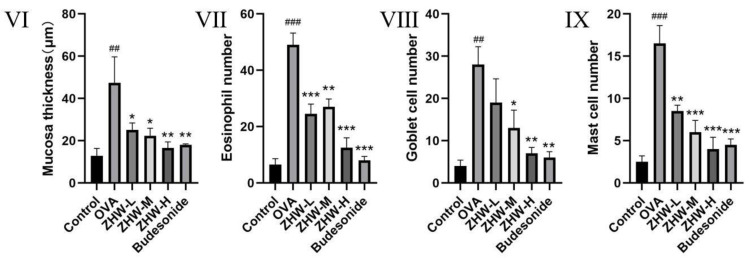
Histopathological evaluation of nasal mucosa and lung tissues in OVA-induced AR mice treated with ZHW. (**I**) Thickness of nasal epithelium assessed by hematoxylin–eosin (H&E) staining. (**II**) Eosinophil infiltration visualized using Giemsa staining (indicated by black arrows). (**III**) Goblet cell hyperplasia highlighted by Periodic acid–Schiff (PAS) staining (black arrows). (**IV**) Migration of mast cells to the nasal epithelium detected via Toluidine blue (TB) staining (red arrows). (**V**) Histological structure of lung tissue analyzed by H&E staining. (**VI**) Nasal mucosa thickness (μm); (**VII**) Eosinophil count; (**VIII**) Goblet cell count; (**IX**) Mast cell count. A: Control group; B: OVA model group; C: Low-dose ZHW group (ZHW-L); D: Medium-dose ZHW group (ZHW-M); E: High-dose ZHW group (ZHW-H); I: Budesonide group. Scale bars: 50 μm for nasal sections and 100 μm for lung sections. Data are presented as mean ± SD (*n* = 6 per group). ANOVA with Bonferroni post hoc test; ## *p* < 0.01, ### *p* < 0.001 vs. control group; * *p* < 0.05, ** *p* < 0.01, *** *p* < 0.001 vs. OVA group.

To confirm the inhibitory effects of ZHW on nasal symptoms and NALF inflammatory cell infiltration in OVA-induced AR mice, we examined the histopathological changes in nasal mucosa and lung tissues. As shown in Figure 7I–VI, H&E staining of nasal mucosa revealed that, compared with the control group, the OVA group exhibited significant interstitial edema (indicated by blue arrows), inflammatory cell infiltration, and a marked increase in mucosal thickness (*p* < 0.01) (Figure 7I). After ZHW treatment, interstitial edema and inflammatory cell infiltration were alleviated, and mucosal thickness decreased in a dose-dependent manner, with significant differences observed in the high-dose group compared with the OVA group (*p* < 0.001). To investigate the infiltration of eosinophils in the nasal mucosa, Giemsa staining was employed. As shown in Figure 7II, inflammatory cell infiltration, including eosinophils (indicated by black arrows), was observed. Compared with the control group, the OVA group had a significant increase in eosinophil numbers (*p* < 0.01), while ZHW treatment led to a marked reduction in eosinophils in all dose groups (*p* < 0.01). PAS staining (Figure 7III) showed significant goblet cell hyperplasia in the OVA group compared with the control group (*p* < 0.01) (indicated by black arrows), which was reduced after ZHW treatment, with significant differences in the high-dose group compared with the OVA group (*p* < 0.01). Toluidine blue staining (Figure 7IV) revealed significant mast cell infiltration in the OVA group compared with the control group (*p* < 0.001). After ZHW treatment, mast cell infiltration was significantly reduced in all dose groups (*p* < 0.01) in a dose-dependent manner.

H&E staining of lung tissue showed that, compared with the control group, OVA-induced AR mice exhibited significant pathological changes in lung tissue, including mucus production in the bronchi, goblet cell hyperplasia, and inflammatory cell infiltration (Figure 7V). After ZHW inhalation treatment, these histopathological changes in lung tissue were alleviated. These results indicate that ZHW has a protective effect on the nasal mucosa of OVA-induced AR mice and can reduce nasal mucosal inflammatory cell infiltration as well as improve pulmonary inflammatory infiltration.

### 2.8. Summary of Protein Expression Profiles Across Different Groups

**Figure 8:**
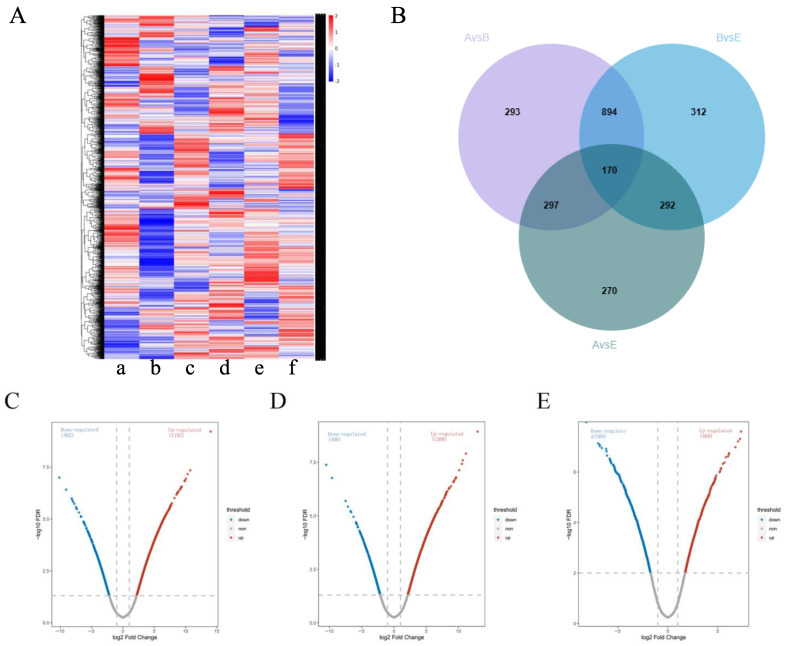
Comparison of protein expression profiles among treatment groups. (**A**) Cluster map comparing the proteins identified in the control group. [a] OVA-induced AR mice, [b] ZHW-L, [c] ZHW-M [d], ZHW-H [e], budesonide. [f] The red color indicates a higher expression (upregulation), blue indicates a lower expression (downregulation), and white indicates similar expression levels. (**B**) Venn diagrams of DEPs among the B/A group, E/B group, and E/A group. (**C**) Volcano plot illustrating protein expression changes in the B/A comparison group. (**D**) Volcano plot showing differential protein distribution in the E/B group. (**E**) Volcano plot displaying expression variations in the E/A group. In panels (**B**–**D**), each plot represents a log2 fold change (FC) on the x-axis against the −log10 *p*-value on the y-axis. Red dots indicate significantly upregulated proteins (*p* < 0.05, log2 FC > 0.58), while blue dots denote non-significant fold changes (*p* < 0.05, |log2 FC| < 0.58).

The DIA analysis identified a total of 5443 quantified proteins, of which 4910 quantified proteins met the quality control requirements after QC correction. By comparing the protein expression profiles among different groups, it was found that the protein expression profiles of group A/B were significantly different from those of the other groups; however, group A/E showed a more similar pattern (Figure 8A), indicating that high-dose ZHW treatment could significantly alter the protein expression profile of AR mice, bringing it closer to that of the normal control group.

In the B/A, E/B, and A/E groups, 1654, 1668, and 1029 proteins were identified as differentially expressed proteins (DEPs), respectively (Figure 8B–E). Among them, 1064 DEPs were common between the B/A and E/B groups, while 170 DEPs were present in all three groups.

### 2.9. Biological Processes and Pathway Changes Between the OVA-Induced Group and the Control Group (B/A Group)

**Figure 9:**
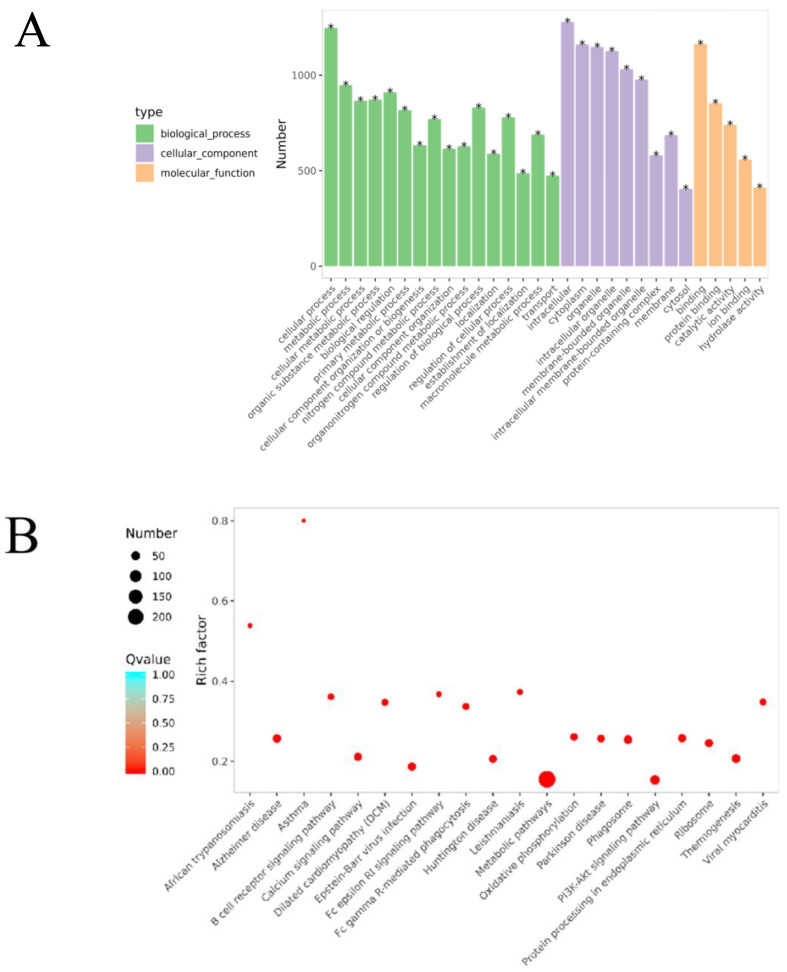
Bioinformatics analysis of the differentially expressed proteins in the OVA-induced group and control group (B/A Group). (**A**) The top 30 categories of enriched GO analysis associated with DEPs, including biological process, molecular function, and cellular component. * FDR < 0.05 (**B**) The top 20 categories of KEGG-enriched pathways associated with DEPs. Rich factor represents the enrichment factor and reflects the enrichment degree. Number indicates the number of enriched proteins. The larger the bubble, the more different proteins are detected. The value range of the Q value is [0,1], and the smaller the value, the more significant the enrichment degree.

GO functional enrichment analysis revealed that the DEPs in the B/A group were mainly associated with biological processes (BPs) such as cellular processes, metabolic processes, and responses to stimuli (Figure 9A, Tables S1 and S2), indicating significant changes in cellular metabolic activities and responses to environmental stimuli in OVA-induced AR mice. Cellular component (CC) analysis showed that DEPs were primarily located in the intracellular space, cytoplasm, and mitochondria, suggesting that these organelles may play key roles in the pathogenesis of AR. In terms of molecular function (MF), DEPs were mainly involved in protein binding and catalytic activity, and changes in these functions may affect cellular signaling and metabolic regulation.

KEGG pathway enrichment analysis identified that these DEPs were involved in several pathways, including the PI3K-Akt signaling pathway, the FcεRI signaling pathway, oxidative phosphorylation, and the B cell receptor signaling pathway (Figure 9, Table S3). Specifically, 58 DEPs were involved in the PI3K-Akt signaling pathway, with 46 upregulated and 12 downregulated. In the oxidative phosphorylation pathway, 40 DEPs were identified, with 33 upregulated and 7 downregulated. The FcεRI signaling pathway included 33 DEPs, of which 12 were upregulated and 21 were downregulated. The B cell receptor signaling pathway had 27 DEPs, with 11 upregulated and 16 downregulated.

These results indicate that in the OVA-induced AR mouse model, significant changes occurred in key signaling pathways such as the PI3K-Akt signaling pathway, oxidative phosphorylation pathway, FcεRI signaling pathway, and B cell receptor signaling pathway. These changes may play important roles in the pathogenesis of AR. Abnormal activation or inhibition of these pathways is closely related to the activation and migration of inflammatory cells and the release of inflammatory mediators, thereby leading to inflammation of the nasal mucosa and the manifestation of AR symptoms.

### 2.10. Biological Processes and Pathway Changes Between the ZHW-H Group and the OVA-Induced Group (E/B Group)

**Figure 10:**
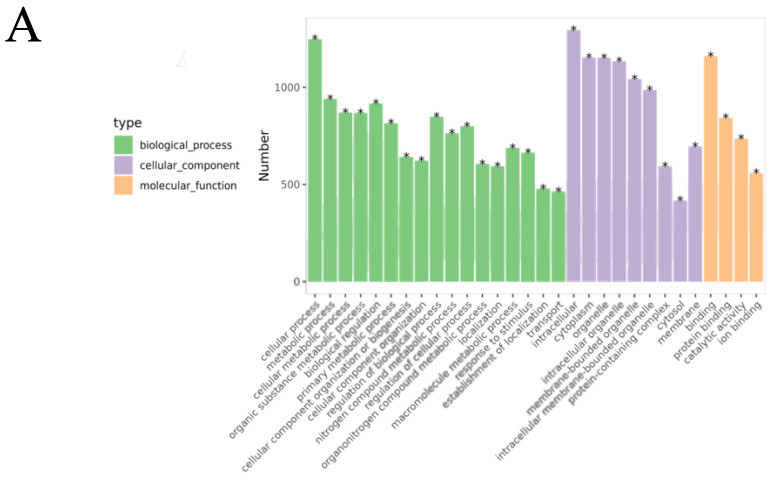

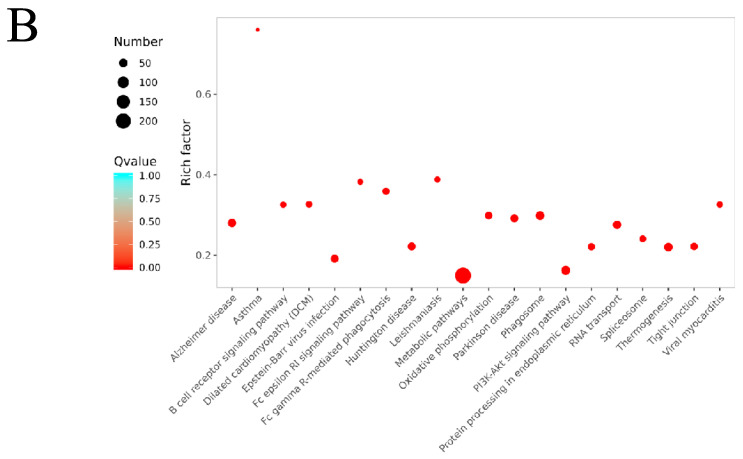
Bioinformatics analysis of the differentially expressed proteins in the ZHW-H related group and the OVA-induced group (E/B group). (**A**) The top 30 categories of enriched GO analysis associated with DEPs, including biological process, molecular function, and cellular component. * FDR < 0.05 (**B**) The top 20 categories of KEGG-enriched pathways associated with DEPs. Rich factor represents enrichment factor and reflects enrichment degree. Number indicates the number of enriched proteins. The larger the bubble, the more different proteins are detected. The value range of the Q value is [0,1], and the smaller the value, the more significant the enrichment degree.

**Figure 11:**
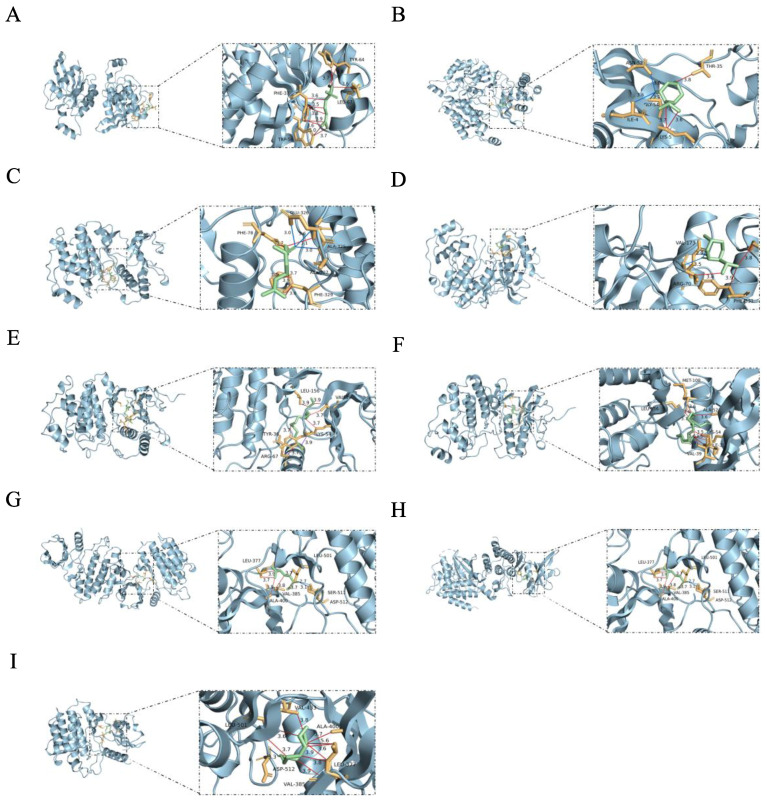
Molecular docking results of active components with core targets. The blue, red, and purple lines represent hydrogen bonds, hydrophobic interactions, and salt bridge interactions, respectively. (**A**) RAC1—linalool, −5.0 kcal/mol. (**B**) RAC1—levomenthol, −5.3 kcal/mol. (**C**) MAPK1—linalool, −5.3 kcal/mol. (**D**) MAPK1—levomenthol, −5.3 kcal/mol. (**E**) MAPK1—linoleic acid, −5.0 kcal/mol. (**F**) MAPK1—Linoelaidic acid, −5.3 kcal/mol. (**G**) SYK—linalool, −5.4 kcal/mol. (**H**) SYK—levomenthol, −5.0 kcal/mol. (**I**) SYK—n-Valeric acid cis-3-hexenyl ester, −5.1 kcal/mol.

The ZHW-H group and the OVA-induced group shared 1668 DEPs. The associated biological processes and pathways were similar to those in the B/A group, mainly involving cellular processes, metabolic processes, and cellular metabolic processes (Figure 10A, Tables S4–S6), as well as the PI3K-Akt signaling pathway, the FcεRI signaling pathway, oxidative phosphorylation, and the B cell receptor signaling pathway (Figure 11B, Table S7). Specifically, 55 DEPs were involved in the PI3K-Akt signaling pathway, with 42 upregulated and 13 downregulated; 35 DEPs participated in oxidative phosphorylation, with 29 upregulated and 6 downregulated; 25 DEPs were involved in the FcεRI signaling pathway, with 22 upregulated and 3 downregulated; and the B cell receptor signaling pathway included 30 DEPs, of which 24 were upregulated and 6 were downregulated.

Proteomic analysis revealed significant differential expression between the ZHW-H and OVA-induced groups, with enriched proteins predominantly involved in core biological processes, including cellular metabolism, immune regulation, and inflammatory control. The marked alterations observed in the PI3K-Akt cascade, the oxidative phosphorylation system, FcεRI-mediated signaling, and the B cell receptor pathway collectively demonstrate that ZHW exerts its therapeutic effects on allergic rhinitis through multi-pathway modulation, effectively mitigating both inflammatory responses and clinical allergic manifestations in the murine model.

### 2.11. Reversal Analysis of Proteins Between the B/A and E/B Groups

To gain a deeper understanding of the mechanisms underlying the effects of ZHW, we compared and analyzed the reversal of protein expression between the OVA-induced group and the control group (B/A group), as well as between the high-dose ZHW treatment group and the OVA-induced group (E/B group).

In this study, we identified several key proteins that were upregulated more than 100-fold in the B/A group, such as Rac1, the IgM heavy chain VDJ region, IgG1 TS1 VH, and Ighv1–42, with Ighv1–42 reaching an upregulation of 268-fold. In addition to these, proteins like MAPK1 and SYK were also identified, all of which are involved in the PI3K-Akt signaling pathway, B cell receptor signaling pathway, and Fc epsilon RI signaling pathway. These pathways play crucial roles in the pathogenesis of AR. Moreover, MAPK1 and SYK, as key proteins in these pathways, are also significantly involved in the pathogenesis of AR. MAPK1 is involved in the regulation of cell proliferation and differentiation and inflammatory responses [17], while SYK plays a key role in the activation and signaling of immune cells [18]. Abnormal activation or upregulation of these proteins is closely related to the pathological process of AR.

In the PI3K-Akt signaling pathway, there were 37 reversed proteins (RPs) between the B/A and E/B groups. Specifically, in the B (OVA)/A (control) group, 28 RPs were significantly upregulated, and 9 RPs were significantly downregulated. In contrast, these RPs exhibited a markedly opposite trend in the E/B group. In addition to proteins such as Rac1, IgG1 TS1 VH, and Ighv1–42, the RPs in the PI3K-Akt signaling pathway also included Itgb3, Angpt2, Gnb2, Itgb1, Lama3, Rps6, Ighv6-6, Syk, Itga6, Map2k2, Gng12, Gm45713, Chuk, Gnb1, Kras, Cdkn1b, Ywhag, Mapk1, Tnr, Ppp2r2a, and Spp1. The PI3K-Akt signaling pathway is vital for cell survival and proliferation, metabolism, and inflammatory responses. Its activation is closely related to the overproduction of Th2 cytokines, which can promote the activation and migration of inflammatory cells, thereby exacerbating inflammation of the nasal mucosa. Moreover, this pathway regulates the secretion of Th2 cytokines, such as IL-4, further intensifying the inflammatory response [19].

The oxidative phosphorylation pathway provides energy support for the activation and proliferation of immune cells, especially during inflammatory responses, when immune cells require a substantial amount of energy to maintain their functions [20]. Analysis revealed a total of 28 RPs, with 23 DEPs upregulated and 5 DEPs downregulated in the B/A group. In contrast, these proteins exhibited an opposite trend in the E/B group. Atp5mg was upregulated by 374-fold in the B/A group but was significantly downregulated in the E/B group. This protein is a subunit of ATP synthase involved in energy metabolism and oxidative phosphorylation. Other RPs in this pathway included Sdha, Sdhc, Ndufv3, Ndufs4, Ndufa3, Ndufv3, Ndufb3, Cox6c, and Atp5f1e. Abnormal activation of this pathway may lead to disordered energy metabolism, thereby exacerbating inflammatory responses and tissue damage.

The B cell receptor signaling pathway plays a key role in the activation of B cells and antibody production [21]. Its activation promotes the differentiation of B cells into plasma cells, increasing the production of IgE, which leads to the overactivation of mast cells and basophils, resulting in the release of inflammatory mediators such as histamine. A total of 20 RPs were identified, with 17 upregulated and 3 downregulated in the B/A group, and an opposite trend was found in the E/B group. RPs in the B cell receptor signaling pathway included Kras, Mapk1, Map2k2, Rac1, Ighv6-6, and Syk.

The FcεRI signaling pathway is initiated by the interaction between antigens and IgE [22]. Activated mast cells release granules containing histamine and cytokines such as IL-4, which exacerbate inflammatory responses and lead to an imbalance of Th1/Th2 cells [23]. A total of 16 RPs were identified, with 14 DEPs upregulated and 2 DEPs downregulated in the B/A group, and an opposite trend was found in the E/B group. RPs in this pathway also included Syk, Map2k2, Rac2, Kras, Mapk1, and Map2k6.

After high-dose ZHW treatment, the expression of these proteins was significantly downregulated or upregulated, returning to levels close to those of the normal control group. These results suggest that ZHW may exert its therapeutic effects on AR by regulating the expression of these key proteins, thereby affecting the activity of the PI3K-Akt signaling pathway, B cell receptor signaling pathway, and FcεRI signaling pathway. This leads to the inhibition of inflammatory cell infiltration, a reduced production of IgE, and a decreased release of inflammatory mediators. Ultimately, ZHW alleviates the symptoms of AR. By downregulating these key proteins, ZHW restores the balance of Th1/Th2 cytokines and mitigates the symptoms of allergic rhinitis. In conclusion, ZHW shows significant potential in the treatment of allergic rhinitis, with its multi-pathway and multi-target regulatory mechanisms effectively alleviating OVA-induced AR symptoms.

### 2.12. Analysis of Molecular Docking Results

Based on the comprehensive analysis of network pharmacology and proteomics, we identified AMPK1, SYK, and RAC1 as key targets. We selected l-Menthone and linalool, which are the most abundant components in ZHW, as well as linoleic acid, Linoelaidic acid, and cis-3-hexenyl valerate, which were predicted by network pharmacology to contribute the most to AR, for molecular docking experiments. The experimental results showed that the binding energies of these components with the targets were all ≤−5.0 kcal/mol, indicating stable binding. The specific data are shown in Figure 11. By stably binding to AMPK1, SYK, and RAC1, these components may inhibit the activation and migration of inflammatory cells and reduce the release of inflammatory mediators, thereby alleviating the symptoms of allergic rhinitis.

## 3. Discussion

This study systematically evaluated the therapeutic effects and potential mechanisms of the traditional Chinese medicine compound ZHW on AR through an innovative inhalation administration method, combined with network pharmacology, proteomics, and animal experiments. The results indicate that ZHW significantly alleviates OVA-induced AR symptoms by regulating differentially expressed proteins in key signaling pathways, demonstrating good therapeutic potential.

The innovation of ZHW lies in its unique inhalation administration method. Compared with traditional oral administration, inhalation can directly act on the nasal mucosa, taking effect quickly, reducing the metabolic process of the drug in the body, increasing the local concentration of the drug, and thereby enhancing the therapeutic effect [24]. Traditional oral administration often leads to reduced drug bioavailability due to the first-pass effect of the liver, while inhalation bypasses hepatic first-pass metabolism, reduces potential impacts on other organs, increases drug bioavailability, and is particularly suitable for children and the elderly. Moreover, ZHW adds *Houttuynia cordata* Thunb. and *Citrus reticulata* Blanco. to the base of “Cang-Er-Zi-San,” enhancing anti-inflammatory properties, improving the olfactory experience, and increasing patient compliance.

Animal experimental results show that ZHW significantly reduces the frequency of sneezing and nose scratching in OVA-induced AR mice, lowers the levels of IL-4, HIS, and sIgE in serum, and alleviates nasal mucosal inflammation. While the effect size varied across groups, these results suggest that ZHW has potential therapeutic benefits that warrant further exploration. These results are consistent with the changes in differentially expressed proteins and pathways found through network pharmacology and proteomics analysis. Specifically, proteomics analysis shows that the expression of key proteins such as Rac1, the IgM heavy chain VDJ region, IgG1 TS1 VH, Ighv1–42, MAPK1, and SYK changed significantly after ZHW treatment. Rac1, a small GTPase, is mainly involved in cytoskeletal remodeling and cell migration and is closely related to the infiltration of inflammatory cells [25]. After ZHW treatment, the expression of Rac1 was significantly downregulated, indicating that ZHW can inhibit the infiltration of inflammatory cells. The IgM heavy chain VDJ region, IgG1 TS1 VH, and Ighv1–42 represent the heavy chain variable regions of IgM and IgG1 antibodies, which are closely related to B cell activation and IgE production. After ZHW treatment, the expression of these proteins was significantly downregulated, indicating that ZHW can inhibit B cell activation and IgE production [26]. MAPK1 and SYK are key proteins in the PI3K-Akt signaling pathway and FcεRI signaling pathway, involved in the activation and signaling of inflammatory cells. After ZHW treatment, the expression of these proteins was significantly downregulated, indicating that ZHW can inhibit the activation of these signaling pathways and reduce the release of inflammatory mediators.

Quantitative dose–response analysis revealed heterogeneous patterns across different parameters. Progressive dose-dependent improvements were observed in behavioral outcomes (nasal rubbing), immune responses (lymphocytes, serum IFN-γ), and tissue remodeling (goblet cells, mucosal thickness). However, several inflammatory markers, including serum IL-4, histamine, OVA-sIgE, and mast cell infiltration, achieved plateau effects at medium doses (1.0 g), suggesting a therapeutic ceiling around this concentration. Notable exceptions included sneezing frequency, which showed non-linear U-shaped responses, and eosinophil infiltration, which exhibited biphasic patterns, likely reflecting complex multi-target interactions characteristic of herbal formulations. Threshold-type responses were observed in thymus index and NALF cytokines, requiring higher doses for significance. These heterogeneous dose–response profiles indicate that while 1.0 g provides optimal benefit for systemic inflammatory control, 2.0 g may be necessary for maximal local tissue effects and behavioral symptom relief.

Network pharmacology and proteomics analysis further revealed the impact of ZHW on key signaling pathways. ZHW significantly affected the PI3K-Akt signaling pathway, oxidative phosphorylation pathway, B cell receptor signaling pathway, and FcεRI signaling pathway. The PI3K-Akt signaling pathway is pivotal for cell survival and proliferation, metabolism, and regulation of inflammatory responses [27]. By downregulating the expression of key proteins such as MAPK1, ZHW inhibited the activation of the PI3K-Akt signaling pathway, reducing the activation and migration of inflammatory cells. The B cell receptor signaling pathway plays a key role in B cell activation and antibody production [28]. By downregulating the expression of proteins such as the IgM heavy chain VDJ region, IgG1 TS1 VH, and Ighv1–42, ZHW inhibited the activation of the B cell receptor signaling pathway, reducing IgE production. The Fc epsilon RI signaling pathway is initiated by the interaction between antigens and IgE, with activated mast cells releasing inflammatory mediators such as histamine [15]. By downregulating the expression of key proteins such as SYK, ZHW inhibited the activation of the FcεRI signaling pathway, reducing the release of inflammatory mediators.

Molecular docking analysis further confirmed the stable binding interactions between the active components of ZHW and their potential therapeutic targets. The results show that the binding energies of the main components of ZHW (such as menthol, linalool, linoleic acid, trans-linoleic acid, and pentyl pentanoate) with key targets (such as AMPK1, SYK, and RAC1) were all ≤−5.0 kcal/mol, indicating stable binding. These results are consistent with the analysis of network pharmacology and proteomics, further supporting the multi-pathway and multi-target regulatory mechanisms of ZHW in exerting anti-allergic rhinitis effects. In particular, linalool, menthol, linoleic acid, trans-linoleic acid, and pentyl pentanoate play a key role in the activity against AR, providing an important theoretical basis for the further development and clinical application of ZHW.

Notably, beyond its anti-inflammatory properties, linalool, the major constituent of ZHW, exhibits significant analgesic effects. Studies have demonstrated that linalool possesses antinociceptive activity in various pain models, with analgesic mechanisms involving opioid receptors, cholinergic receptors, and the PI3K/Akt signaling pathway [29,30]. This finding is highly consistent with the PI3K/Akt signaling pathway identified in our study, suggesting that linalool may simultaneously exert anti-inflammatory and analgesic effects through modulation of shared molecular networks. Furthermore, linalool odor exposure can activate hypothalamic orexinergic neurons through the olfactory pathway, subsequently inhibiting spinal pain signal transmission [31]. This directly supports the rationale for ZHW’s inhalation administration route—enabling both local anti-inflammatory effects and systemic pain relief through the olfactory–neural pathway to alleviate AR-related nasal pain and discomfort.

This comprehensive preclinical evaluation demonstrates ZHW’s significant therapeutic potential through validated multi-target mechanisms. The innovative inhalation delivery method, combined with the multi-pathway regulatory effects, positions ZHW as a promising candidate for AR treatment. However, we acknowledge that linalool and levomenthol, major components of ZHW, have been reported to cause allergic contact dermatitis upon dermal exposure [32,33,34]. In addition to skin sensitization, certain low-molecular-weight compounds may also induce respiratory sensitization via the nasal mucosa. Sugiura et al. [35] reported a significant increase in positive patch test reactions to lavender oil in Japan, possibly related to its widespread use in aromatherapy and other consumer products. As linalool is a common component of both lavender oil and ZHW, this epidemiological observation highlights the potential sensitization risks associated with frequent exposure to natural volatile substances. Furthermore, Aimen et al. [36] reviewed animal models showing that repeated inhalation of certain low-molecular-weight chemicals can elicit airway inflammation and elevated IgE levels, indicative of respiratory allergic responses. In contrast, under the inhalation conditions used in our study, no signs of hypersensitivity were observed, including eosinophilic nasal inflammation, Th2 cytokine elevation, or increased IgE. Although the components of ZHW differ structurally from typical chemical sensitizers and no immunotoxic or sensitizing effects were detected, the potential for nasal sensitization under long-term or repeated exposure should not be overlooked. Future studies should include targeted inhalation safety assessments to further verify the long-term immunological safety of ZHW.

## 4. Materials and Methods

### 4.1. Preparation of ZHW

The raw materials of ZHW were all purchased from Lingnan Chinese Medicinal Materials Co., Ltd. (Foshan, China), and glycerol was purchased from Shanghai Aladdin Biochemical Technology Co., Ltd. (Shanghai, China). ZHW is composed of six common medicinal materials, namely *Xanthium sibiricum* Patr. (Lot Number: 190915008), *Magnolia denudata* Desr. (Lot Number: 180613062), *Angelica dahurica* (Lot Number: 200914001), *Houttuynia cordata* Thunb. (Lot Number: 170912061), *Citrus reticulata* Blanco. (Lot Number: 200112074), and *Mentha haplocalyx* Briq. (Lot Number: 200512040) (fresh).

Preparation process: Based on an in-depth study of the optimal inhalation administration method, the optimal proportion of each medicinal material in ZHW was determined to be 2:2:3:4:4:2. The preparation procedure was as follows: The six medicinal materials were crushed and passed through a 100-mesh sieve and mixed evenly. Then, the glycerol solution was slowly added to the medicinal material powder and stirred until the mixture could be lightly squeezed into a mass and would disintegrate upon touch. After granulation through a No. 2 sieve and drying, the mixture was cooled and then sized through a No. 16 sieve to remove oversized or undersized particles, yielding the final product with uniform particle size.

### 4.2. Gas Chromatography–Mass Spectrometry (GC-MS) Analysis

Gas chromatography–mass spectrometry equipment (GC System, Agilent HP-5MS Ultra Inert, Agilent Technologies, Santa Clara, CA, USA) equipped with a DB-5 quartz capillary column (30 m × 250 μm × 0.25 μm) was employed to analyze ZHW. The testing conditions were as follows: The carrier gas was high-purity helium (purity 99.999%) with a flow rate of 1 mL/min; the injection port temperature was set at 220 °C, and the injection volume was 1 μL (splitless injection). The temperature program was as follows: The initial temperature was held at 40 °C for 1 min, then increased to 100 °C at a rate of 5 °C/min and held for 2 min, subsequently raised to 190 °C at a rate of 3 °C/min and held for 3 min, and finally increased to 260 °C at a rate of 8 °C/min and held for 2 min. The ion source temperature was maintained at 230 °C, and the electron ionization mode (70 eV) was used with the full-scan mode. The chemical constituents in ZHW were identified by comparing the spectra with those recorded in the NIST mass spectral library.

### 4.3. Network Pharmacology Analysis

Network pharmacology is an emerging interdisciplinary research approach that aims to systematically analyze the mechanisms of drug action by constructing complex networks between drugs, targets, and diseases. This method helps us understand the multi-target effects of drugs and reveals the complex interactions between drugs and biological systems.

#### 4.3.1. Target Prediction

Potential targets of drug components are predicted through database searches and computational tools (such as Swiss Target Prediction). These targets are the proteins or genes that drugs can bind to and exert biological effects on.

In this study, the effective components of ZHW were identified by combining gas chromatography–mass spectrometry (GC-MS) analysis results with the China National Knowledge Infrastructure (CNKI) database. Subsequently, the SMILES notations of these components were retrieved from the PubChem database (https://pubchem.ncbi.nlm.nih.gov/, accessed on 8 November 2023) and imported into the Swiss Target Prediction database (http://swisstargetprediction.ch/index.php, accessed on 8 November 2023) for target prediction, selecting targets with prediction probabilities greater than 0. The predicted component targets were then screened and summarized, with duplicates removed, to obtain the drug targets of ZHW. Thereafter, the Online Mendelian Inheritance in Man (OMIM, https://omim.org/, accessed on 14 November 2023), GeneCards (http://www.genecards.org/, accessed on 14 November 2023), and DrugBank (https://www.drugbank.ca/, accessed on 14 November 2023) databases were utilized to screen for targets related to allergic rhinitis (AR) using “allergic rhinitis” as the keyword.

#### 4.3.2. Network Construction

The drug components were connected with their predicted targets to construct the drug–target network. By analyzing the topological structure of the network, key targets and drug components could be identified.

A Venn diagram of the intersection between the effective components of ZHW and AR targets was constructed using Venny 2.1.0 (https://bioinfogp.cnb.csic.es/tools/venny/, accessed on 16 November 2023), and these component–target data were imported into Cytoscape 3.10.0 software (https://www.cytoscape.org, accessed on 20 November 2023) to construct a network of the effective components of ZHW and their targets.

Subsequently, the intersection targets were uploaded to the STRING database (https://string-db.org/, accessed on 27 November 2023), where “Multiple proteins” was selected, the species was set to “Homo sapiens,” and the interaction score was set to 0.4 for protein interaction analysis to obtain the interactions between targets. The TSV file generated by the STRING platform was saved and imported into Cytoscape 3.10.0 software to construct a target network. Topological analysis was then performed to obtain relevant data for the core target network and construct a core target protein–protein interaction (PPI) network.

#### 4.3.3. Gene Ontology Function and KEGG Pathway Enrichment Analysis

Gene Ontology (GO) is a standardized system of biological terms used to describe gene functions, which includes three aspects: the molecular function (MF), the activity of gene products at the molecular level (e.g., enzyme activity, receptor binding); biological process (BP), the biological processes in which genes are involved (e.g., inflammatory response, immune regulation); and cellular component (CC), the cellular structure or location where gene products are found (e.g., cell membrane, nucleus). In addition to GO analysis, we also performed KEGG pathway analysis to investigate the biological pathways involved in the predicted targets of ZHW. This helps to reveal the potential mechanisms of drug action.

The intersection targets were imported into the DAVID 6.8 database (https://david.ncifcrf.gov/, accessed on 29 November 2023), with the species defined as “Homo sapiens,” for Kyoto Encyclopedia of Genes and Genomes (KEGG) pathway enrichment analysis and Gene Ontology (GO) function enrichment analysis to reveal the potential pathways of ZHW against allergic rhinitis. Finally, the association data between targets and pathways were imported into Cytoscape 3.10.0 software to construct a network of potential targets and pathways of ZHW against allergic rhinitis, achieving visualization of the target–pathway network.

### 4.4. Animal Experimental Methods

In this experiment, purebred Kunming mice of PF grade, female, 4 weeks of age, and weighing 18–22 g were used. The experimental animals were provided by Hubei Bentech Biotechnology Co., Ltd. and were approved by the laboratory animal care committee of Wuhan Polytechnic University (WPU202301003). The certificate number for the experimental animals is NO. 422023100002802, and the permit number is SCXK (Hubei) 2021-0027. The breeding conditions were as follows: The experimental mice were housed in separate cages with free access to food and drinking water, with a 12 h light/dark cycle, good ventilation in the room, a breeding temperature maintained at 21–25 °C, and a relative humidity of 50–70%. After a one-week acclimatization period to allow the mice to adapt to the laboratory environment (light/dark cycle, temperature, humidity, and handling), the animals were randomly assigned into six experimental groups, with nine mice in each group (n = 9): the control group, the OVA group, the low-dose ZHW group (ZHW-L, 0.5 g), the medium-dose ZHW group (ZHW-M, 1.0 g), the high-dose ZHW group (ZHW-H, 2.0 g), and the budesonide group (1.0 mg).

#### 4.4.1. OVA-Induced Animal Model and Treatment Methods

**Figure 12:**
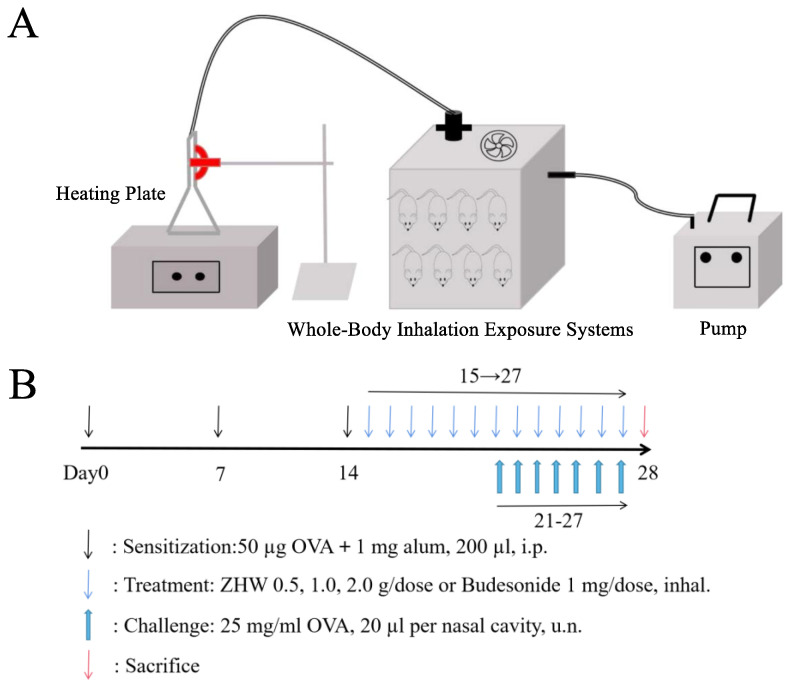
Experimental design and protocol for mouse modeling and drug administration. (**A**) Inhalation administration device for mice. (**B**) Schematic diagram of experimental group design and protocol for OVA-induced allergic rhinitis mouse model.

OVA was purchased from Shanghai Yuanye Biotechnology Co., Ltd. (Shanghai, China). Normal saline was purchased from Qingzhou Yaoyu Pharmaceutical Co., Ltd. (Weifang, China). Budesonide suspension was purchased from AstraZeneca Pty Ltd. (Macquarie, Australia). As shown in Figure 12B, the experimental methods were as follows: Except for the control group, the other five groups of mice were sensitized systemically by intraperitoneal injection of 200 μL PBS containing 50 μg OVA and 1 mg aluminum hydroxide adjuvant on days 0, 7, and 14, while the control group mice were injected with an equal volume of PBS at the same time points. From day 21 to day 27, once daily, the mice were locally sensitized by intranasal administration of 20 μL of 25 mg/mL OVA solution to each nostril [37]. The treatment protocol was as follows: From day 15 to day 27, all mice were simultaneously treated with the corresponding drugs. Specifically, the heating plate temperature was set at 260 °C, and the mice were placed in the whole-body inhalation exposure system for 20 min. The traditional Chinese medicine compound ZHW was administered via an inhalation device (Figure 12A), which ensured complete animal exposure to the vapor generated by ZHW volatile oils at three dose levels: low dose (ZHW-L, 0.5 g), medium dose (ZHW-M, 1.0 g), and high dose (ZHW-H, 2.0 g). Specifically, 0.5 g, 1.0 g, or 2.0 g of ZHW granules were placed on the heating plate for each treatment group (n = 9 mice per group), with all mice in each group exposed simultaneously to the generated vapor. The budesonide group received the drug via nebulization at a dose of 1 mg, once daily. The control group was given an equal volume of glycerol for inhalation as a control. The experimental endpoint was as follows: on day 28, after the last drug administration, the mice were sacrificed for subsequent detection.

#### 4.4.2. Body Weight and Behavioral Tests

Nasal symptoms were evaluated by calculating the frequency of nasal rubbing and sneezing. On the last day of the OVA challenge, the number of sneezes and nasal rubs in each group of mice was recorded blindly within 20 min after OVA stimulation. Meanwhile, the body weight of each group of mice was recorded over six days from day 22 to day 27.

#### 4.4.3. Peripheral White Blood Cell Count in Mice

On day 28, whole blood from mice was collected via cardiac puncture and placed into 2 mL EDTA-coated tubes. Subsequently, 50 μL of the anticoagulated blood sample was taken and analyzed using an automated hematology analyzer (model: BC-30Vet) to determine the total number of white blood cells and the proportion of each component. The detection indicators included WBC, Gran, Lymph, and Mon.

The EDTA-coated tubes were purchased from Shijiazhuang Kangweishi Medical Device Co., Ltd. (Shijiazhuang, China).

#### 4.4.4. Thymus and Spleen Indices of Mice

After orbital blood collection, thymus and spleen tissues were harvested from mice in each group. Excess blood was blotted from the tissues using filter paper before weighing. The thymus and spleen indices were calculated using the following formulas: Thymus index = Thymus weight (mg)/Mouse body weight (g), Spleen index = Spleen weight (mg)/Mouse body weight (g).

### 4.5. Enzyme-Linked Immunosorbent Assay (ELISA)

Four hours after the last OVA challenge, blood was collected from the mice via retro-orbital sinus puncture. The collected blood was centrifuged at 3000× *g* for 10 min at 4 °C, and the supernatant, i.e., serum, was collected and stored in a −80 °C freezer for later use. After the mice were sacrificed, a tube was placed into the trachea in the direction of the nasal cavity, and the nasal cavity was flushed with 1 mL of normal saline to collect the nasal lavage fluid (NALF) supernatant, which was also stored in a −80 °C freezer for later use. The levels of cytokines IFN-γ, IL-4, HIS, and OVA-sIgE in the serum and NALF were detected using ELISA kits.

The ELISA kits for mouse IFN-γ, IL-4, HIS, and OVA-sIgE were all purchased from Shanghai Kebio Biotechnology Co., Ltd. (Shanghai, China).

### 4.6. Histopathological Analysis

The mouse nasal tissues were fixed in 10% neutral formalin at room temperature for 7 days, followed by decalcification for 4 weeks, and then subjected to paraffin embedding, sectioning (with a thickness of 4 μm), and dewaxing. The sections were stained with H&E, TB, PAS, and Giemsa, respectively. H&E staining was used to examine the overall morphology of the nasal mucosa and lung tissues, TB staining was employed to detect the infiltration of mast cells in the nasal mucosa, PAS staining was utilized to assess the infiltration of goblet cells and mucus in the nasal mucosa, and Giemsa staining was applied to quantify and evaluate the infiltration of eosinophils.

### 4.7. Quantitative Proteomics Analysis

#### 4.7.1. Protein Extraction and Peptide Digestion

Five mice from each group were randomly selected for proteomic analysis, and their protein tissues were stored in liquid nitrogen for subsequent use. For the remaining mice in each group, their nasal mucosa tissues were taken out and thawed on ice. For each sample, 0.03 g of tissue was placed into a 1.5 mL grinding tube, to which 150 μL of 4% sodium dodecyl sulfate (containing PMSF) was added. The grinding tube was then placed in a grinder and ground for 10 min, followed by heating the ground sample in a dry bath at 90 °C for 10 min. After removal, the sample was ground again for another 10 min. Finally, the sample was centrifuged at 13000 rpm for 10 min, and the supernatant, i.e., the protein solution, was collected. The protein concentration of each sample was measured using a BCA protein assay kit. Thirty-five micrograms of protein extract were subjected to gel electrophoresis to observe the distribution of proteins. One hundred micrograms of protein were taken from each sample for enzymatic digestion, and the peptides were extracted and collected, then dried in a vacuum centrifuge and stored at 4 °C for subsequent LC-MS/MS analysis.

Sodium dodecyl sulfate and trypsin were purchased from Beijing Solarbio Science & Technology Co., Ltd. (Beijing, China), and the BCA protein assay kit was purchased from Shanghai Beyotime Biotechnology Co., Ltd. (Shanghai, China).

#### 4.7.2. DDA Library Data Acquisition Preparation

After enzymatic digestion of the peptide samples, equal amounts were taken from each biological sample and mixed. A total of 200 μg of the mixture was subjected to high-pH reversed-phase (RP) chromatography fractionation. The chromatographic conditions were as follows: an Agilent 1100 series liquid chromatography system (Agilent Technologies, Santa Clara, CA, USA) was used to separate each fractionated sample. The chromatographic separation conditions for the protein samples were as follows: flow rate-0.60 mL/min; injection volume: 100 μL; mobile phase: phase A was 0.1% formic acid in water; phase B was acetonitrile/water (95:5, *v*/*v*); the liquid chromatography elution gradient was 120 min, with phase B increasing from 3% to 100%; 3% phase B as the initial concentration (0 min), 3–7% phase B (0–2 min), 7–22% phase B (2–92 min), 22–40% phase B (92–107 min), 40–100% phase B (107–110 min), and 3% phase B balanced to 120 min. The mass spectrometry conditions were as follows: A Triple TOF 6600 (SCIEX, Framingham, MA, USA) mass spectrometer was used to analyze the samples after chromatographic separation. The analysis lasted for 120 min, with a detection mode of positive ion, a parent ion scan range of 350–1500 *m*/*z*, an AGC target of 400,000, a maximum injection time of 50 ms, a normalized AGC target of 100%, and a dynamic exclusion time of 20.0 s.

#### 4.7.3. DDA Database Construction Data Collection

Proteome Discoverer 2.1.0182 (Thermo Fisher Scientific, Rockford, IL, USA) was used to analyze the LC-MS/MS DDA data obtained after fractionation for protein identification. The search engine employed was Sequest HT, and the protein database used was the UniProt database (TaxaID:10090). Other settings are shown in Table 2.

#### 4.7.4. DIA Quantitative Data Collection

The samples were subjected to DIA data acquisition, and quantitative analysis was performed using Skyline software (version 7.1.0) (Department of Genome Sciences, University of Washington, Ave. NE, Seattle, WA, USA). Detailed parameters are shown in Table 3.

### 4.8. Bioinformatics Analysis

To evaluate the data structure of the identified proteins in the biological samples and the distribution of proteins among the samples, we performed hierarchical clustering analysis using the quantified protein data. Additionally, we conducted functional annotation and analysis of the identified differentially expressed proteins through Gene Ontology (GO), Kyoto Encyclopedia of Genes and Genomes (KEGG) pathway analysis, and protein–protein interaction (PPI) network analysis. The GO annotation covered three main aspects: biological process (BP), cellular component (CC), and molecular function (MF).

### 4.9. Molecular Docking

Molecular docking was performed using AutoDock Vina software (https://vina.scripps.edu/) to dock the key target structures from proteomics with the structures of active components. The docking was carried out using Vina within PyRx 0.9 software. The affinity values (kcal/mol) represent the binding capabilities between the active components and the key targets. Visualization analysis was conducted using PyMOL 4.6 software.

### 4.10. Statistical Analysis

The results are expressed as the mean ± standard deviation (SD) of the number of experiments. Statistical analysis was performed using ANOVA with a Bonferroni post hoc test. A *p*-value < 0.05 was considered to indicate statistical significance.

## 5. Conclusions

In conclusion, ZHW significantly alleviates OVA-induced AR symptoms through the stable binding of multiple components, including levomenthol, linalool, linoleic acid, Linoelaidic acid, and n-Valeric acid cis-3-hexenyl ester, with key targets such as Rac1, MAPK1, and SYK. This effect is primarily mediated by the PI3K-Akt signaling pathway, B cell receptor signaling pathway, FcεRI signaling pathway, and oxidative phosphorylation pathway. These findings not only elucidate the molecular mechanisms underlying ZHW’s anti-allergic effects but also provide a robust scientific foundation for developing novel AR therapeutics. The demonstrated multi-pathway modulation offers a template for modernizing traditional Chinese medicine formulations through mechanism-based optimization. Future research will further explore the clinical potential of ZHW and validate its therapeutic efficacy and safety through additional experiments and clinical trials.

Future research will focus on clinical efficacy trials accompanied by a comprehensive safety evaluation to establish the complete risk–benefit profile of ZHW for AR treatment.

## Figures and Tables

**Table 1:** Identified compounds in ZHW volatile oil.

Number	Retention Time (s)	Compounds	Molecular Formula	CAS	Area Sum%
1	9.401	Sabinene	C10H16	003387-41-5	0.55
2	10.855	p-Cymene	C10H14	000099-87-6	0.44
3	11.061	Eucalyptol	C10H18O	000470-82-6	4.43
4	11.861	gamma-Terpinene	C10H16	000099-85-4	0.25
5	13.281	Linalool	C10H18O	000078-70-6	17.69
6	13.504	Maltol	C6H6O3	000118-71-8	0.4
7	14.691	Isopulegol	C10H18O	000089-79-2	1.18
8	14.975	D-isomenthone	C10H18O	001196-31-2	4.29
9	15.044	Menthone	C10H18O	000089-80-5	3.83
10	15.431	l-Menthone	C10H18O	014073-97-3	6.25
11	16.025	Levomenthol	C10H20O	002216-51-5	24.64
12	16.274	Dl-menthol	C10H20O	001490-04-6	0.47
13	16.721	Dodecane	C12H26	000112-40-3	0.47
14	17.134	Neoisomenthol	C10H20O	000491-02-1	0.3
15	17.96	Citronellol	C10H20O	000106-22-9	1.42
16	18.038	5-Hydroxymethylfurfural	C6H6O3	000067-47-0	0.86
17	18.227	n-Valeric acid cis-3-hexenyl ester	C11H20O2	035852-46-1	0.35
18	18.382	Para-menth-4(8)-en-3-one	C10H16O	015932-80-6	1.44
19	18.984	Piperitone	C10H16O	000089-81-6	1.26
20	19.053	Geraniol	C10H18O	000106-24-1	1.58
21	19.569	Cinnamaldehyde	C9H8O	000104-55-2	0.36
22	20.644	Menthyl acetate	C12H22O2	000089-48-5	3.2
23	21.358	2-Methoxy-4-vinylphenol	C9H10O2	007786-61-0	0.3
24	21.47	Decadienal	C10H16O	025152-84-5	0.33
25	23.027	cis-2,6-Dimethyl-2,6-octadiene	C10H18	002492-22-0	0.34
26	24.601	γ-Elemene	C15H24	110823-68-2	0.38
27	25.693	Caryophyllene	C15H24	000087-44-5	0.79
28	27.053	(Z)-β-Ocimene	C10H16	003338-55-4	0.36
29	28.162	Germacrene D	C15H24	023986-74-5	0.22
30	28.799	α-Amorphene	C15H24	000483-75-0	0.31
31	29.461	gamma-Muurolene	C15H24	030021-74-0	0.21
32	29.848	δ-Cadinene	C15H24	000483-76-1	0.54
33	30.855	Elemol	C15H26O	000639-99-6	0.64
34	32.79	Hexadecane	C16H34	000544-76-3	0.22
35	34.812	alpha-Cadinol	C15H26O	000481-34-5	0.3
36	39.981	Octadecane	C18H38	000593-45-3	0.23
37	45.418	n-Hexadecanoic acid	C16H32O2	000057-10-3	0.57
38	50.459	Linoelaidic acid	C16H32O2	000506-21-8	0.87
39	50.579	Linolic acid	C16H32O2	000060-33-3	0.57

**Table 2:** Setup parameters of Proteome Discoverer 2.1.0182.

Item	Parameter
Enzyme	Trypsin
Miss Cleavages	2
Variable Modifications	Oxidation (M), Acetyl (Protein N-term), Deamidated (N, Q)
Fixed Modifications	Carbamidomethyl (C)
Peptide Mass Tolerance	± 10 ppm
Fragment Mass Tolerance	± 0.02 Da
Peptide FDR	Less than 1%
Protein Q Value	Less than 1%

**Table 3:** Detailed parameters of Skyline software.

Items	Parameter Settings
Peptide length	6–25
Sub-ion type	B, y
Sub-ion *m*/*z* selection	>parent ion and last ion-3
Maximum number of daughter ions	5
Maximum number of daughter ions	2

## Data Availability

The data presented in this study are available on request from the corresponding author.

## Supplementary Materials

The following supporting information can be downloaded at: https://www.mdpi.com/article/10.3390/ph18071059/s1, Table S1. 15 important biological process (BP) in GO analysis in the B/A group. Table S2. 15 important cellular component (CC) in GO analysis in the B/A group. Table S3. KEGG pathways associated with the DEPs in the B/A group. Table S4. 15 important biological process (BP) in GO analysis in the E/B group. Table S5. 15 important cellular component (CC) in GO analysis in the E/B group. Table S6. KEGG pathways associated with the DEPs in the E/B group. Tabel S7. Reversed Proteins in PI3K-Akt signaling pathway. Tabel S8. Reversed Proteins in Oxidative phosphorylation. Tabel S9. Reversed Proteins in Fc epsilon RI signaling pathway. Tabel S10. Reversed Proteins in B cell receptor signaling pathway.

## Abbreviations

AR	Allergic Rhinitis
OVA	Ovalbumin
OVA-sIgE	Ovalbumin-specific Immunoglobulin E
KM-Kunming	Kunming
IL-4	Interleukin-4
Th1	T Helper 1
Th2	T Helper 2
TLR4	Toll-Like Receptor 4
MCs	Mast Cells
EOSs	Eosinophils
IFN-γ	Interferon-gamma
TCM	Traditional Chinese Medicine
Gran	Granulocytes (Neutrophils, Eosinophils, Basophils)
Lymph	Lymphocytes
Mon	Monocytes
NALF	Nasal Lavage Fluid
HIS	Histamine
SDS	Sodium Dodecyl Sulfate
DEPs	Differentially Expressed Proteins
RPs	Reversed Proteins
IgE	Immunoglobulin E
IgM	Immunoglobulin M
PI3K-Akt	Phosphoinositide 3-Kinase–Protein Kinase B
Rac1	Ras-related C3 Botulinum Toxin Substrate 1
MAPK1	Mitogen-Activated Protein Kinase 1
SYK	Spleen Tyrosine Kinase
FcεRI	High-affinity IgE Receptor (Fc Epsilon RI)
