# Supplementary material for: Therapeutic Effects and Mechanisms of the Inhaled Traditional Chinese Medicine Compound ZHW on Allergic Rhinitis
Source: Pharmaceuticals (Basel). 2025 Jul 18;18(7):1059. doi: 10.3390/ph18071059 (PMC12300735; doi:10.3390/ph18071059)
Supplement: Supplementary file 1 [file pharmaceuticals-18-01059-s001.zip › pharmaceuticals-3721106-supplementary.pdf]

Supplementary Materials

The differentially expressed proteins in the OVA-induced Group and Control Group (B/AGroup)

Table S1 15 important biological process (BP) in GO analysis in the B/A group

GO Term	GO ID	Count	P Value
cellular process	GO:0009987	1248	2.55E-249
metabolic process	GO:0008152	940	4.67E-160
cellular metabolic process	GO:0044237	870	3.68E-145
organic substance metabolic process	GO:0071704	867	2.07E-135
biological regulation	GO:0065007	916	3.14E-130
primary metabolic process	GO:0044238	815	8.45E-120
cellular component organization or biogenesis	GO:0071840	641	2.38E-117
cellular component organization	GO:0016043	623	1.34E-113
regulation of biological process	GO:0050789	848	3.47E-112
nitrogen compound metabolic process	GO:0006807	763	2.73E-107
regulation of cellular process	GO:0050794	799	2.92E-103
organonitrogen compound metabolic process	GO:1901564	605	1.05E-97
localization	GO:0051179	593	2.83E-96
macromolecule metabolic process	GO:0043170	687	3.92E-84
response to stimulus	GO:0050896	663	6.96E-82

Table S2 15 important cellular component (CC) in GO analysis in the B/A group

GO Term	GO ID	Count	P Value
intracellular	GO:0005622	1294	3.73E-311
cytoplasm	GO:0005737	1153	1.55E-284
organelle	GO:0043226	1151	1.45E-242
intracellular organelle	GO:0043229	1134	2.98E-241
membrane-bounded organelle	GO:0043227	1043	7.03E-208
intracellular membrane-bounded organelle	GO:0043231	986	3.61E-194
protein-containing	GO:0032991	594	2.61E-127

complex			
cytosol	GO:0005829	417	5.12E-95
membrane	GO:0016020	695	9.40E-94
organelle membrane	GO:0031090	299	1.40E-75
nucleus	GO:0005634	572	5.16E-73
intracellular	GO:0043232	426	7.15E-72
non-membrane-bounded organelle			
non-membrane-bounded organelle	GO:0043228	427	8.02E-72
mitochondrion	GO:0005739	274	2.76E-71
endomembrane system	GO:0012505	406	1.57E-68

Table S3 KEGG pathways associated with the DEPs in the B/A group

Term	ID	Input number	Background number	P-Value
Metabolic pathways	mmu01100	223	1494	1.76E-50
Phagosome	mmu04145	54	181	2.67E-24
Alzheimer disease	mmu05010	49	175	3.43E-21
RNA transport	mmu03013	46	167	9.65E-20
Parkinson disease	mmu05012	42	144	6.53E-19
Oxidative phosphorylation	mmu00190	40	134	2.25E-18
Thermogenesis	mmu04714	51	232	3.93E-18
Fc gamma R-mediated phagocytosis	mmu04666	33	92	2.43E-17
Huntington disease	mmu05016	43	194	1.27E-15
Dilated cardiomyopathy (DCM)	mmu05414	31	95	1.92E-15
PI3K-Akt signaling pathway	mmu04151	58	358	4.07E-15
Asthma	mmu05310	19	25	5.25E-15
Leishmaniasis	mmu05140	26	67	1.28E-14
Viral myocarditis	mmu05416	29	89	1.59E-14

Fc epsilon RI signaling pathway	mmu04664	26	68	1.70E-14
Epstein-Barr virus infection Epstein-Barr	mmu05169	44	230	5.67E-14
Tight junction	mmu04530	37	167	1.22E-13
B cell receptor signaling pathway	mmu04662	27	83	1.31E-13
Protein processing in endoplasmic reticulum	mmu04141	36	163	2.83E-13
Spliceosome	mmu03040	32	133	8.22E-13

The differentially expressed proteins in ZHW-H reated Group and OVA-induced Group (E/B Group)

Table S4 15 important biological process (BP) in GO analysis in the E/B group

GO Term	GO ID	Count	P Value
cellular process	GO:0009987	1247	5.52E-244
metabolic process	GO:0008152	948	4.85E-162
cellular metabolic process	GO:0044237	866	9.45E-141
organic substance metabolic process	GO:0071704	872	7.72E-136
biological regulation	GO:0065007	911	2.14E-125
primary metabolic process	GO:0044238	818	2.50E-119
cellular component organization or biogenesis	GO:0071840	633	1.08E-111
nitrogen compound metabolic process	GO:0006807	770	7.26E-109
cellular component organization	GO:0016043	615	4.92E-108
organonitrogen compound metabolic process	GO:1901564	628	1.09E-107
regulation of biological process	GO:0050789	831	2.79E-102
localization	GO:0051179	589	4.79E-93
regulation of cellular process	GO:0050794	779	1.73E-92

establishment of localization	GO:0051234	488	1.85E-84
macromolecule metabolic process	GO:0043170	689	1.53E-83

Table S5 15 important cellular component (CC) in GO analysis in the E/B group

GO Term	GO ID	Count	P Value
intracellular	GO:0005622	1278	1.09E-292
cytoplasm	GO:0005737	1161	1.48E-286
organelle	GO:0043226	1148	1.60E-236
intracellular organelle	GO:0043229	1127	1.54E-232
membrane-bounded organelle	GO:0043227	1033	2.83E-198
intracellular membrane-bounded organelle	GO:0043231	976	4.32E-185
protein-containing complex	GO:0032991	580	4.07E-118
membrane	GO:0016020	686	3.14E-88
cytosol	GO:0005829	405	1.56E-87
endomembrane system	GO:0012505	429	1.14E-78
intracellular non-membrane-bounded organelle	GO:0043232	430	7.46E-73
non-membrane-bounded organelle	GO:0043228	431	5.76E-69
nucleus	GO:0005634	565	4.06E-67
organelle membrane	GO:0031090	285	8.45E-73
mitochondrion	GO:0005739	263	1.83E-64

Table S6 KEGG pathways associated with the DEPs in the E/B group

Term	ID	Input number	Background number	P-Valu e
Metabolic pathways	mmu01100	232	1494	9.09E-55
Phagosome	mmu04145	46	181	1.82E-18
Alzheimer disease	mmu05010	45	175	2.90E-18
Protein processing in endoplasmic reticulum	mmu04141	42	163	3.62E-17
Dilated	mmu05414	33	95	6.36E-17

cardiomyopathy (DCM)				
Ribosome	mmu03010	43	175	6.68E-17
Thermogenesis	mmu04714	48	232	3.78E-16
Viral myocarditis	mmu05416	31	89	5.19E-16
Asthma	mmu05310	20	25	5.68E-16
B cell receptor signaling pathway	mmu04662	30	83	6.78E-16
Fc gamma R-mediated phagocytosis	mmu04666	31	92	1.10E-15
Parkinson disease	mmu05012	37	144	2.91E-15
Oxidative phosphorylation	mmu00190	35	134	1.07E-14
Calcium signaling pathway	mmu04020	41	194	2.86E-14
African trypanosomiasis	mmu05143	21	39	3.42E-14
Leishmaniasis	mmu05140	25	67	9.73E-14
Huntington disease	mmu05016	40	194	1.18E-13
Fc epsilon RI signaling pathway	mmu04664	25	68	1.28E-13
PI3K-Akt signaling pathway	mmu04151	55	358	1.86E-13
Epstein-Barr virus infection	mmu05169	43	230	2.68E-13

Reversed Proteins between OVA-induced Group and Control Group (B/A Group) and ZHW-H reated Group and OVA-induced Group (E/B Group).

Tabel S7 Reversed Proteins in PI3K-Akt signaling pathway

NO.	Protein Name	Gene Name	Uniprot Assention	B/A	E/B
1	Integrin beta-3	Itgb3	O54890	4.88	0.18
2	Angiopoietin-2	Angpt2	O35608	5.39	0.19
3	Guanine nucleotide	Gnb2	E9QKR0	6.4	0.13

	binding protein (G protein), beta 2				
4	Integrin beta-1	Itgb1	P09055	6.42	0.12
5	Laminin subunit alpha-3	Lama3	Q61789	6.45	0.09
6	40S ribosomal protein S6	Rps6	Q5BLK1	7.95	0.16
7	Immunoglobulin heavy variable 6-6	Ighv6-6	A0A075B5T3	8.07	0.05
8	Tyrosine-protein kinase	Syk	Q3UPF7	8.91	0.15
9	Itga6 protein	Itga6	Q80ZI8	9.51	0.14
10	Immunoglobulin heavy variable V1-20	Ighv1-20	A0A0A6YX66	10.00	0.03
11	Dual specificity mitogen-activated protein kinase kinase 2	Map2k2	Q63932	10.07	0.12
12	Guanine nucleotide-binding protein subunit gamma	Gng12	A0A0N4SW28	10.73	0.17
13	60S ribosomal protein L13a	Gm45713	A0A1B0GS68	11.01	0.19
14	Inhibitor of nuclear factor kappa-B kinase subunit alpha	Chuk	E9QNL4	12.79	0.16
15	Beta1 subnuit of GTP-binding protein	Gnb1	Q3TQ70	14.14	0.05
16	GTPase KRas	Kras	P32883	14.81	0.14
17	Cyclin-dependent kinase inhibitor 1B	Cdkn1b	P46414	15.11	0.06
18	14-3-3 protein gamma subtype	Ywhag	A8IP69	16.12	0.18
19	Mitogen-activated protein kinase 1	Mapk1	P63085	18.54	0.09
20	Tenascin-R	Tnr	Q8BYI9	22.3	0.02
21	Ig-like domain-containing protein	------	Q9D8L4	25.93	0.03
22	Ig heavy chain V region RF	------	P18524	30.02	0.06
23	Serine/threonine-protein phosphatase 2A 55 kDa regulatory subunit B	Ppp2r2a	Q571J7	34.84	0.02
24	Secreted phosphoprotein 1	Spp1	F8WIP8	49.69	0.04

25	Rac family small GTPase 1	Rac1	Q3TLP8	108.53	0.01
26	IgM heavy chain VDJ region	------	X5J5N3	114.61	0.01
27	Gamma heavy chain variable region	IgG1 TS1 VH	Q5F2I8	129.7	0.11
28	Immunoglobulin heavy variable V1-42	Ighv1-42	A0A075B5V6	268.84	0.02
29	Ephrin type-A receptor 2	Epha2	Q03145	0.02	19.13
30	Fab4201 heavy chain	--------	A0A0M3KL48	0.03	24.57
31	14-3-3 protein eta	Ywhah	P68510	0.035	16.33
32	Immunoglobulin heavy variable V8-12	Ighv8-12	A0A0G2JDE1	0.09	20.81
33	Chondroadherin	Chad	Q3TYW1	0.10	9.18
34	VWFA domain-containing protein	Col6a6	B9EK79	0.12	12.87
35	14-3-3 protein theta	Ywhaq	P68254	0.12	27.75
36	Multifunctional fusion protein	Fgf1	Q6ZWS1	0.14	20.96
37	Growth hormone a1	Prl	Q9CPQ2	0.20	6.17

Tabel S8 Reversed Proteins in Oxidative phosphorylation

NO.	Protein Name	Gene Name	Uniprot Assention	B/A	E/B
1	Succinate dehydrogenase flavoprotein subunit, mitochondrial	Sdha	Q8K2B3	5.44	0.12
2	V-type proton ATPase subunit C	ATP6v1c1	Q3TG21	7.16	0.14
3	NADH dehydrogenase flavoprotein 3, mitochondrial	Ndufv3	Q3U422	7.44	0.17
4	NADH dehydrogenase iron-sulfur protein 4, mitochondrial	Ndufs4	E9QPX3	7.96	0.20
5	NADH dehydrogenase 1 alpha subcomplex subunit 3	Ndufa3	Q9CQ91	8.15	0.15
6	Succinate dehydrogenase cytochrome b560 subunit, mitochondrial	Sdhc	Q5XK33	8.21	0.12
7	NADH dehydrogenase flavoprotein 3,	Ndufv3	Q91WP8	9.13	0.05

	mitochondrial				
8	NADH dehydrogenase 1 beta subcomplex subunit 3	Ndufb3	Q9CQZ6	9.40	0.12
9	Cytochrome c oxidase subunit 6C	Cox6c	Q9CPQ1	11.59	0.07
10	ATP synthase subunit epsilon, mitochondrial	Atp5f1e	P56382	11.96	0.03
11	ATP synthase subunit b	Atp5pb	Q3TJD4	12.25	0.08
12	NADH dehydrogenase flavoprotein 3, mitochondrial	Ndufv3	Q8BK30	12.35	0.11
13	Phospholysine phosphohistidine inorganic pyrophosphate phosphatase	Lhpp	Q9D7I5	12.45	0.01
14	V-type proton ATPase subunit B, kidney isoform	ATP6v1b1	Q91YH6	14.04	0.10
15	NADH-ubiquinone oxidoreductase chain 4	ND4	A0A023J6G6	20.28	0.09
16	Cytochrome c oxidase subunit 5A, mitochondrial	Cox5a	P12787	22.6	0.05
17	NADH dehydrogenase iron-sulfur protein 8, mitochondrial	Ndufs8	Q3UY05	23.81	0.15
18	V-type proton ATPase subunit G	Atp6v1g1	Q5HZY7	24.86	0.03
19	Cytochrome c oxidase subunit 3	COX3	A0A023J659	25.99	0.03
20	Acyl carrier protein	Ndufab1	Q3TJG2	27.97	0.02
21	NADH-ubiquinone oxidoreductase chain 1	ND1	A0A023J609	33.41	0.01
22	NADH dehydrogenase 1 beta subcomplex subunit 8, mitochondrial	Ndufb8	Q9D6J5	41.94	0.04
23	ATP synthase subunit	Atp5l	Q9CQY3	374.73	0.01
24	NADH dehydrogenase 1 alpha subcomplex subunit 7	Ndufa7	A0A068BGR9	0.09	25.13
25	Cytochrome c oxidase subunit 7A1	Cox7a1	A0A140LIU4	0.11	23.58
26	ATP synthase subunit gamma	Atp5c1	Q3UD06	0.18	13.97
27	NADH dehydrogenase [ubiquinone] 1 alpha subcomplex subunit 2	Ndufa2	Q9CQ75	0.19	13.31
28	Inorganic pyrophosphatase	Ppa2	Q91VM9	0.20	8.27

2, mitochondrial

Tabel S9 Reversed Proteins in Fc epsilon RI signaling pathway

NO.	Protein Name	Gene Name	Uniprot Assention	B/A	E/B
1	Immunoglobulin heavy variable 6-6	Ighv6-6	A0A075B5T3	8.07	0.05
2	Tyrosine-protein kinase	Syk	Q3UPF7	8.91	0.15
3	Immunoglobulin heavy variable V1-20	Ighv1-20	A0A0A6YX66	10.00	0.03
4	Dual specificity mitogen-activated protein kinase kinase 2	Map2k2	Q63932	10.07	0.12
5	Ras-related C3 botulinum toxin substrate 2	Rac2	Q05144	13.32	0.10
6	GTPase KRas	Kras	P32883	14.81	0.14
7	Mitogen-activated protein kinase 1	Mapk1	P63085	18.54	0.09
8	Protein kinase domain-containing protein	Map2k6	Q543Z5	25.85	0.01
9	Ig-like domain-containing protein	------	Q9D8L4	25.93	0.03
10	Ig heavy chain V region RF	------	P18524	30.02	0.06
11	Rac family small GTPase 1	Rac1	Q3TLP8	108.53	0.01
12	IgM heavy chain VDJ region	------	X5J5N3	114.61	0.01
13	Gamma heavy chain variable region	IgG1 TS1 VH	Q5F2I8	129.7	0.10
14	Immunoglobulin heavy variable V1-42	Ighv1-42	A0A075B5V6	268.84	0.02
15	Fab4201 heavy chain	------	A0A0M3KL48	0.026	24.57
16	Immunoglobulin heavy variable	Ighv8-12	A0A0G2JDE1	0.09	20.81

	V8-12				

Tabel S10 Reversed Proteins in B cell receptor signaling pathway

NO.	Protein Name	Gene Name	Uniprot Assention	B/A	E/B
1	Immunoglobulin heavy variable V8-12	Ighv8-12	Q6P549	6.26	0.16
2	Immunoglobulin heavy variable 6-6	Ighv6-6	A0A075B5T3	8.07	0.05
3	Tyrosine-protein kinase	Syk	Q3UPF7	8.91	0.15
4	Immunoglobulin heavy variable V1-20	Ighv1-20	A0A0A6YX66	10.00	0.03
5	Dual specificity mitogen-activated protein kinase kinase 2	Map2k2	Q63932	10.07	0.12
6	Inhibitor of nuclear factor kappa-B kinase subunit alpha	Chuk	E9QNL4	12.79	0.16
7	Ras-related C3 botulinum toxin substrate 2	Rac2	Q05144	13.32	0.10
8	GTPase KRas	Kras	P32883	14.81	0.14
9	Mitogen-activated protein kinase 1	Mapk1	P63085	18.54	0.09
10	Fc receptor, IgG, low affinity IIb	Fcgr2b	A0A0B4J1G1	20.34	0.15
11	Ig-like domain-containing protein	-----	Q9D8L4	25.93	0.03
12	PPP3ca protein	PPP3ca	Q4V9X0	26.32	0.08
13	Ig heavy chain V region RF	-----	P18524	30.02	0.06
14	Rac family small GTPase 1	Rac1	Q3TLP8	108.53	0.01
15	IgM heavy chain VDJ region	------	X5J5N3	114.61	0.01
16	Gamma heavy chain variable region	IgG1 TS1 VH	Q5F2I8	129.7	0.10

17	Immunoglobulin heavy variable V1-42	Ighv1-42	A0A075B5V6	268.84	0.02
18	Fab4201 heavy chain	-----	A0A0M3KL48	0.026	24.57
19	Immunoglobulin heavy variable V8-12	Ighv8-12	A0A0G2JDE1	0.09	20.81
20	Protein phosphatase 3 catalytic subunit alpha	Ppp3ca	P63328	0.093	11.97
